# Polish adaptation of ‘Maltreatment and Abuse Chronology of Exposure’ scale

**DOI:** 10.1371/journal.pone.0321046

**Published:** 2025-04-09

**Authors:** Magdalena Chęć, Sylwia Michałowska, Karolina Rachubińska, Krystian Konieczny, Agnieszka Samochowiec

**Affiliations:** Department of Clinical Psychology and Psychoprophylaxis, Institute of Psychology, University of Szczecin, Szczecin, Poland; Universitat der Bundeswehr München: Universitat der Bundeswehr Munchen, Germany

## Abstract

**Background:**

Adverse childhood experiences, such as abuse, maltreatment or neglect, can lead to many mental disorders and emotional and social difficulties.

**Objective:**

The aim of this study was to adapt and validate the ‘Maltreatment and Abuse Chronology of Exposure’ (MACE) questionnaire to Polish socio-cultural conditions.

**Participants and setting:**

The study involved 330 adult, white people (60.8% women); aged between 18 and 86 years (*M* =  41.01; *SD* =  14.67), with and without a psychiatric diagnosis.

**Methods:**

Convergent validity was assessed by comparing MACE (PL) scores with the CTQ (Childhood Trauma Questionnaire) and ACE (Adverse Childhood Experience Questionnaire), while predictive validity was determined by examining the relationships between MACE (PL) scores and the SCL-90 (Symptom Checklist-90). The psychometric properties of the scale were assessed using Rasch analysis, which evaluated item fit, difficulty, and person separation. Internal consistency was measured using the Kuder Richardson coefficient (KR-20). The Polish version of MACE demonstrated good reliability, as indicated by high internal consistency (KR-20) and findings from Rasch analysis.

**Results:**

The Polish version of MACE, after analysis, included a total of 58 items combined into 10 scales. The Polish version of the scale showed high internal consistency, measured using the Kuder-Richardson formula (KR-20). The results of the Polish version of MACE showed strong and positive correlations with the scores of CTQ and ACE. These correlations were particularly evident for the overall MACE scores and subscales such as MACE SUM, MACE Multiplicity, and MACE Duration. Correlations for physical neglect and emotional neglect were moderate but statistically significant (r =  0.49, p <  0.001; r =  0.46, p <  0.001). MACE scores were positively correlated with SCL-90 and ACE results, indicating high predictive validity in relation to psychopathological symptoms. The correlations between MACE and SCL-90 were moderate but significant, suggesting that MACE effectively predicts psychopathological symptoms associated with childhood trauma. Most MACE subscales showed moderate reliability (0.5–0.8), except for the PVA and SEXA subscales, which demonstrated high internal consistency (KR20 >  0.8).

**Conclusion:**

The Polish version of MACE demonstrates solid convergent validity, predictive validity, and psychometric reliability, making it a valuable tool for assessing experiences of maltreatment and neglect during childhood in both research and clinical practice. Assessing the history of adverse childhood experiences using the MACE can provide a more precise understanding of how the type and timing of these experiences influence outcomes. This, in turn, sheds light on the mechanisms underlying health and the common pathways contributing to overlapping symptom spectrums. In summary, the MACE appears to be a valuable tool for clinicians and researchers aiming to retrospectively assess the types, timing, and duration of childhood maltreatment experiences during sensitive developmental periods in adulthood.

## Introduction

The concept of trauma is understood as an event or series of events that a person perceives as physically or emotionally damaging and having a long-term adverse impact on their psychological well-being and emotional and social functioning [1,2] Adverse childhood experiences (ACE) is stressful or potentially traumatic events that children experiences before age 18 years [3]. They constitute a risk factor for the development of psychiatric disorders such as post-traumatic stress disorder [4, 5], depression [6], anxiety disorders [7], sleep disorders [8], schizophrenia [9] and personality disorders – mainly borderline personality disorder [10].

Adverse childhood experiences is one of the causes of internalizing and externalizing disorders and other mental illness [11]. It also has a negative impact on prognosis and response to treatment in adults [12]. Adverse childhood experiences result in a number of psychological consequences that include interpersonal difficulties, lowered self-esteem, increased propensity for substance abuse and increased sensitivity to stress [13, 14]. When these experiences take the form of multiple or chronic interpersonal traumas they are referred to as complex trauma. The available research findings indicate that adverse childhood experiences may lead to this type of trauma, which is associated with neurobiological changes in the central nervous system – mainly in terms of reduced volume in the hippocampus and prefrontal cortex, in the endocrine system that regulates stress, and an exaggerated amygdala response to emotionally negative information [15]. The indicated changes are associated with a decrease in an individual’s cognitive functioning, particularly in memory and information processing [16].

The most common causes of adverse childhood experiences are physical abuse, verbal abuse, sexual abuse and household dysfunction such as domestic violence [17]. One of the main environments in which the phenomena described above occur is the family [18]. Risk factors associated with the possibility of adverse childhood experiences in the home environment include: (1) substance abuse, (2) mental illness in the family, (3) violent treatment of either parent, (4) criminal behaviour, (5) parental separation or divorce [19, 20]. A study on abuse and maltreatment in Polish society indicates that 19% of teenagers in Poland have experienced family violence from adults in their lives, and this percentage has increased to 33% when the experience of physical punishment in the form of spanking was included in the analysis. Of the respondents, 19% experienced psychological violence in the family, 6% – neglect physical, and 13% witnessed domestic violence against an adult or another child in the family [21].

There are several tools to measure adverse childhood experiences that may be traumatic. The most common, besides the *Maltreatment and Abuse Chronology of Exposure (MACE)*, are the *Childhood Trauma Questionnaire* (CTQ) [22–24] and the *Adverse Childhood Experiences Scale* (ACE) [25]. These tools are retrospective in nature and refer to the first 18 years of the subject’s childhood.

In its design, the CTQ contains 28 test items that allow to obtain a score on several scales divided according to the type of maltreatment: physical, psychological and sexual violence; emotional neglect, physical neglect.

The original ACE questionnaire includes 10 test items that allow to obtain and describe the results in terms of categories: abuse (psychological, physical, sexual), household dysfunction (substance abuse, mental illness, mother treated violently, criminal behaviour in household).

Despite the numerous advantages of the two tools described and their widespread use, they have certain limitations in their designs. Both tools described above are characterised by the impossibility of specifying the time at which the reported experiences took place. This means that it is not possible to specify the developmental period in which situations potentially influencing the formation of psychopathology occurred. Additionally, the CTQ and ACE questionnaire contain fewer measures of adverse childhood experiences. None of these tools describe peer violence, which is also associated with the development of mental disorders in the future. Moreover, the CTQ does not include items related to witnessing domestic violence, and the ACE questionnaire only describes violence committed against the mother and the stepmother. The MACE [26] is a tool designed to measure exposure to experiences such as abuse and maltreatment up to the age of 18, with the option of noting at what age the events took place. Originally, the authors developed the MACE-X, which is an experimental version that includes more test items than the final version of the scale – 75 questions on 10 types of maltreatment in childhood/adolescence with the possibility of determining the occurrence of the above abuse and neglect in each of the periods up to the age of 18. Scores are captured on scales relating to emotional neglect, physical neglect, parental physical maltreatment, parental verbal abuse, non-verbal emotional abuse, peer emotional abuse, peer physical bullying, sexual abuse, witnessing violence toward siblings and witnessing interparental violence. In the case of convergent validity, taking into account the CTQ and ACE questionnaire, depending on the research, it is indicated that the correlation coefficient should be between 0.6 and 0.8 [26] or greater then 0.6 [27]. The MACE scale demonstrates strong convergent validity with established measures of childhood adversity. This is evidenced by its high correlation coefficients with the CTQ and ACE questionnaire, which fall within the recommended range. Such robust correlations suggest that the MACE scale effectively captures similar constructs of childhood maltreatment and adverse experiences as these well-validated instruments [26].

Based on the MACE-X version, researchers around the world are making adaptations of the tool to the conditions of selected countries and regions. It was created for this moment the Chinese version [28], the Norwegian version [27], the French version [29] and the German version [30]. The adaptations, as well as the original version, are characterised by good psychometric properties, such as test-retest reliability, predictive validity, convergent validity. In addition to the mentioned validations of MACE, there is also a Brazilian Portuguese version that has only been translated into native language [31].

The main aim of the study was to conduct a Polish adaptation of the Maltreatment and Abuse Chronology of Exposure Scale (MACE). The specific aims were:

1. To create a Polish version of the MACE-X based on the American version of the questionnaire, using a back-translation procedure.
2. Construct subscales of the scale based on simple Rasch models. The Rasch model, based on Item Response Theory (IRT), enables the assessment of the reliability of a measurement tool by analyzing the fit of participants’ responses to a probabilistic model. Reliability can be evaluated through the fit of test items to the model (Item Fit), using Infit and Outfit statistics. Infit assesses the consistency of responses near an individual’s ability level, while Outfit evaluates the fit for responses that are less aligned with a given item. Additionally, an analysis of item difficulty was conducted. Item Information Curves illustrate the amount of information provided by a given test item at different ability levels. Higher information values indicate greater measurement precision. Similarly, the test information function represents the total amount of information across various ability levels, with higher values reflecting more accurate measurement.
3. Assessment of the reliability of the Polish version of MACE using the Kuder-Richardson method.
4. To examine the convergent validity of the Polish version of the MACE by assessing its correlations with the Adverse Childhood Experiences (ACE) questionnaire and the Childhood Trauma Questionnaire (CTQ).
5. To investigate the predictive validity of the Polish adaptation of the MACE by analyzing its correlations with the SCL-90 subscales, while simultaneously examining the predictive validity of ACE and CTQ in relation to the SCL-90 dimensions.

## Materials and methods

### Participants

Participants in the study were patients of psychiatric wards and healthy individuals from the general population who were willing to complete the questionnaires. Inclusion criteria for the study included being over 18 years of age and having sufficient knowledge of the Polish language, adequate cognitive ability and mental health to complete the entire questionnaire. Patients hospitalised in the Day Unit and the closed Wards of the Department of Psychiatry of the Clinical University Hospital in North-West Poland and patients staying in the psychiatric Wards of the Independent Public Specialist Institute of Health Care were selected for the clinical groups. Participants were recruited from the general population using a snowball sampling method. Recruitment began with individuals known to the researchers, who were then asked to invite others from their social networks to participate in the study. This approach was chosen to facilitate access to a diverse group of participants that might have been challenging to reach through conventional sampling methods. All participants provided informed consent, and their responses were collected anonymously to ensure confidentiality.

A total of 330 subjects participated in the study, of whom 21 were excluded due to the inability to calculate MACE (incomplete sheets, not respond to questions about traumatic experiences), aged between 18 and 86 years (*M* =  41.01; *SD* =  14.67). The group of subjects included in the analyses consisted of 101 subjects (32.7%) from the clinical group and 206 subjects (66.7%) from the general population. Two of the study participants were still undergoing psychiatric evaluation. The primary diagnoses (according to International Classification of Diseases, 10th Revision [32] given by the clinicians concerned 101 participants and included depression (n = 38), anxiety disorders and phobias (n = 12), schizophrenia (n = 9), bipolar affective disorder (n = 8), personality disorders (n = 7) – predominantly borderline personality disorder (n = 5), alcohol or drug abuse (n = 4), obsessive-compulsive disorder (n = 3), psychotic disorders (n = 3), eating disorders (n = 2) and post traumatic stress disorder (n = 1). Some study participants had more than one psychiatric diagnosis (n = 14). While some participants indicated a leading diagnosis, not all of them did so. This overlap and variability in reporting reflect the presence of comorbid conditions and differing diagnostic emphases.

The majority of the study participants were women (60.8%), living in cities with a population between 100,000 and 500,000 (38.5%). The largest proportion of subjects were married or single, came from a complete family and had siblings. 21.7% had a family history of mental illness. Addiction was present in 14.2% of the subjects, and 26.5% of the subjects reported a family history of addiction. The detailed characteristics of the sample are presented in Table 1.

**Table 1:** Sample characteristics (*N* = 309).

Variables	Statistics
Gender identity, *n (%)*	
Female	187 (60.5%)
Male	121 (39.2%)
Other	1 (0.3%)
Place of residence, *n (%)*	
Village	66 (21.4%)
City up to 50,000 inhabitants	53 (17.2%)
City of 50,000–100,000 inhabitants	38 (12.3%)
City with 100,000–500,000 inhabitants	119 (38.5%)
City with more than 500,000 inhabitants	31 (10.0%)
Missing data	2 (0.6%)
Age, *M (SD)*	
M (SD)	41.01 (14.67%)
18–25	61 (21.7%)
26–35	31 (10.0%)
36–45	54 (17.5%)
46–55	83 (26.9%)
56–65	24 (7.8%)
66–75	9 (2.9%)
76–86	4 (1.3%)
Missing	37 (12.0%)
Marital status, *n (%)*	
Married	136 (44.0%)
Widowed	9 (2.9%)
Single	113 (36.6%)
Divorced	28 (9.1%)
In an informal relationship	20 (6.5%)
Missing data	3 (1.0%)
Mental illness, *n (%)*	
Yes	101 (32.7%)
No	206 (66.7%)
Missing data	2 (0.6%)
Family history of mental illness, *n (%)*	
Yes	67 (21.7%)
No	238 (77.0%)
Missing data	4 (1.3%)
Addictions, *n (%)*	
Yes	44 (14.2%)
No	261 (84.5%)
Missing data	4 (1.3%)
Addictions in the family, *n (%)*	
Yes	82 (26.5%)
No	222 (71.8%)
Missing data	5 (1.6%)

### Development of the adapted questionnaire

The process of adapting the MACE questionnaire for Polish participants occurred in two stages. First, the MACE-X scale was translated, and appropriate items were selected. In the second stage, the reliability and validity of the resulting 58-item scale were evaluated.

The MACE-X, originally consisting of 75 items, measures the severity of exposure to ten types of maltreatment and abuse before the age of 18. These include emotional and physical neglect, verbal and non-verbal emotional abuse, physical and sexual abuse, and witnessing interparental or sibling violence. Negative peer behaviors such as emotional abuse and physical bullying are also assessed. The questionnaire asks respondents to mark ‘YES’ or ‘NO’ for each item, as well as indicate the years during which the abuse or maltreatment occurred (ages 1–18). Some items also ask whether the respondent felt powerless or scared at the time. Eight items referring to positive experiences are reversed during analysis based on the timing of the events, rather than just a simple ‘yes-no’ reversal. This approach ensures that the timing context of each experience is appropriately accounted for in the analysis. The original MACE scale demonstrates adequate reliability and excellent convergent and convergent validity [26].

### Translation process

In August 2022, permission was granted to adapt the MACE for use in Poland. The test items were translated into Polish by two independent psychologists with expertise in childhood violence and English studies. A professional English translator then conducted back-translation to ensure accuracy, followed by another Polish - English translation based on reference materials.

### Selection of relevant items for the Polish version of the MACE

A Polish version of the questionnaire was created, maintaining the original design, including spaces to mark event chronology and to provide relevant content when necessary. The experimental version was tested for clarity and understanding in a pilot study involving 20 participants (11 women, 9 men, average age 25). Ten participants had no psychiatric diagnoses and ten had various mental health diagnoses, including depression, Attention Deficit Hyperactivity Disorder, bipolar disorder, and anorexia. Participants rated their understanding of each item on a scale of 1 (completely not understood) to 5 (completely understood). Items rated below 5 were reviewed and suggestions for clarification were incorporated. Additionally, a YES/NO prompt was used to identify any stigmatizing language, and necessary revisions were made to enhance clarity and avoid stigmatization. Details of the changes made are presented in Table 2.

**Table 2:** Changes made to test items based on respondents’ assessment.

Original content of the MACE test item (PL)	Content of the MACE test item as modified according to the respondents’ suggestions (PL)
“Grożono Ci odejściem lub porzuceniem.”	“Grożono zostawieniem lub porzuceniem Ciebie.” Change made: change of the word order.
“Dotykano Cię lub ***poddawano pieszczotom o charakterze seksualnym”*** (dotyczy dorosłych osób zamieszkujących w domu z osobą badaną).	“Dotykano Twoje ciało ***z podtekstem seksualnym.***” Change made: the term “pieszczoty” has an overly positive connotation to be used in the context being assessed.
“***Próbowano*** odbyć z Tobą stosunek seksualny (oralny, analny lub waginalny).”	***“Usiłowano*** odbyć z Tobą jakikolwiek stosunek seksualny (oralny, analny, waginalny).” Change made: “usiłowano” reflects the forceful nature of this experience more than “próbowano”.
“Dotykano Cię lub ***poddawano pieszczotom o charakterze seksualnym”*** (dotyczy osób dorosłych niezamieszkujących w domu z osobą badaną).	“Dotykano Twoje ciało ***w sposób seksualny.***” Change made: the term “pieszczoty” has an overly positive connotation to be used in the context being assessed.
“Czułeś/-aś, że Twoja matka lub inna ważna dla Ciebie kobieta pełniąca rolę matki była obecna w domu, jednak pozostawała dla Ciebie niedostępna emocjonalnie, z różnych powodów, takich jak nadużywanie narkotyków lub alkoholu, pracoholizm, romanse, czy też dążenie do realizacji własnych celów.”	“Czułeś/-aś, że Twoja matka lub inna ważna dla Ciebie kobieta, pełniąca rolę matki była obecna w domu, jednak pozostawała dla Ciebie niedostępna emocjonalnie z różnych powodów, takich jak używanie narkotyków lub alkoholu, pracoholizm, romanse, czy też ***beztroskie*** skupienie się na własnych celach.” Change made: the term “beztroskie” conveys the focus on the self as conveying the essential meaning of the context being assessed.
“Musiałeś/-aś mieszkać w dwóch lub więcej domach.”	“Musiałeś/-aś ***równocześnie*** mieszkać w dwóch lub więcej domach.” Change made: addition of the term “równocześnie” to emphasise that the question relates to living in multiple houses at the same time and not over time.
“Celowo wykluczano Cię z aktywności lub ***towarzystw.***”	“Celowo wykluczano Cię z aktywności lub ***grup.***” Change made: as suggested by respondents, the word “towarzystw” was replaced by “grup”, which is the word more commonly used in this context in Polish.

Source: own elaboration.

### Assessment of reliability and validity

In September 2022, the Ethical Committee for Research Projects of Institute of Psychology in North-West Poland gave its approval for the study to be conducted as an adaptation of the MACE-X tool.

Subjects from clinical groups and the general population took part in the study. The study took place using the paper-and-pencil method and, in addition to the MACE-X questionnaire in the Polish version, the CTQ, ACE and SCL-90 and author-developed sociodemographic questionnaire was used. The study was conducted by a team of psychologists who work in clinical settings. Graduate students supported the study process by encouraging and supporting patients before, during and after the study, under the supervision of a psychologist. Participants in the clinical group completed the questionnaires in therapy rooms under the supervision of a psychologist. Similarly, individuals from the general population, who volunteered to take part, also completed the questionnaires under the supervision of a psychologist. Prior to the study, participants were verbally informed about its purpose (tool adaptation), data anonymization, and the option to withdraw from the study at any time without providing a reason. Each test sheet was accompanied by a consent form, outlining the scope of the study and the confidentiality of the data. Participants confirmed their consent by initialing the provided information. Completed questionnaires were placed in sealed envelopes, which were opened later by the researchers for data coding. Recruitment took place from April 4, 2023, to November 30, 2023.

#### Comparative measures.

In addition to the MACE-X questionnaire in the Polish language version, the CTQ, ACE and SCL-90 were used in the study, as well as a self-administered questionnaire.

The 10-item *Adverse Childhood Experiences (ACE) Scale* [33] was used, which measures the amount of a person’s exposure to emotional, physical and sexual abuse in childhood and also identifies problem areas in the home, i.e., substance abuse by family members, mental illness, aggressive behaviour towards the mother or stepmother, criminal record of family members or parental separation, divorce or death. The patient’s task is to tick the answer “yes” when a certain event occurred in childhood. The higher the score on the test, the more adverse childhood experiences the person has had. The ACE has good reliability and validity [25,34].

*The Childhood Trauma Questionnaire (CTQ)*, by Bernstein and Fink [22,35], in the Polish version by Murzyn [24], consists of 28 items, through which it is possible to assess a person’s exposure to maltreatment by other people and its form in five categories: physical and emotional abuse, sexual abuse and physical and emotional neglect. The subject is asked to rate the items on a five-point Likert scale (from “never” to “very often”). The survey can be used to provide a total score, which indicates exposure to adverse childhood experiences, and scores on individual scales reflecting different forms of maltreatment and neglect. The CTQ is characterised by sufficient psychometric properties [24].

One of the world’s most popular questionnaires, *the Symptom Checklist-90-R* [36] in the Polish language version by Jankowski, was used to measure general mental health, covering a broad spectrum of psychopathological symptoms. There are at least two translations of the SCL-90-R in Poland, as well as its shortened version, the SCL- 27 [37]. For the research presented in this article, the authors selected a more recent version of the scale. The possibility of using the original SCL questionnaires in Poland has not yet been legally regulated. The subject is asked to rate the extent to which he/she has experienced 90 problems from the list in the past week by marking answers on a 5-point Likert scale from “not at all” to “very much”. These mental health problems fall into nine groups of clinical symptoms: somatization, obsessive -compulsive, interpersonal sensitivity, depression, anxiety, hostility, phobic anxiety, paranoid ideation and psychoticism dimensions.

A self-administered questionnaire was created in order to obtain basic sociodemographic data about the subjects. Its authors also included questions about the financial condition of the family of origin and current family, illnesses or mental disorders of the respondent and family members, as well as questions about the presence of addictions.

### Statistical analysis

Based on questions from the original 75-item MACE-X [26], test items were initially assigned to 10 subscales, in line with the Norwegian, Chinese, and American versions. A simple Rasch model was then specified for each subscale, aiming to include at least 4 test items that measure a given dimension. Items included in the scale were identified based on mean-square fit criteria (χ^2^/df) as measured by infit and outfit. Infit represents responses to items with a level of difficulty (where “difficulty” refers to the “severity of exposure”) that closely matches the individual’s level of exposure, while outfit is more sensitive to observations that deviate significantly from the individual’s level of exposure. Values of 0.5–1.5 were used as acceptable fit thresholds [38]. High mean-square fits were treated more restrictively, as they are more threatening to the overall fit (modelling randomness) than low square fits (indicating overly predictable values). Interpretation of the fit measures started with outfit each time, followed by infit.

The test’s information function was plotted for each subscale, with which the precision of the test in terms of exposure levels was estimated. The peak of the overall information function around logit scores between 0 and 2, i.e., discriminating scores at moderate to higher levels, was taken as the most optimal discrimination of event exposures.

After optimisation based on matching items to subscales, global tests were performed using the Andersen likelihood ratio (LR). LR test indicates the extent to which the data are consistent across subgroups. The analyses used age (split according to the median), gender and the presence of mental illness as the dividing criteria. When the LR test was found to be statistically significant, an additional Wald test was performed after Andersen’s test to identify which test items were responsible for intergroup differences. It allows to check whether the item difficulty parameter differs significantly between subgroups.

We applied Item Response Theory (IRT) to model the relationship between latent traits and observed item responses. IRT was chosen for its ability to account for differences in item difficulty and discrimination, providing more precise estimates of participants’ underlying traits and ensuring the reliability of the scales employed in this study.

The reliability of MACE was estimated using the KR-20 (Kuder-Richardson Formula 20) coefficient. The KR-20 is a measure of internal consistency reliability for tests or assessments that have dichotomous items (items scored as either 0 or 1, such as true/false or correct/incorrect). KR-20 reliability coefficients of less than 0.50 are considered low, between 0.50 and 0.80 moderate, and above 0.80 high [39].

Convergent validity comparing the Polish version of the MACE with the CTQ and ACE was estimated using Pearson correlation coefficients, with the expectation that convergent validity would manifest itself as correlation coefficients >  0.6.

Based on the cut-off thresholds of clinically relevant exposure levels for the CTQ, the ability of the MACE subscales to predict the absence and presence of clinically relevant levels of the CTQ subscales was estimated. To define clinically relevant exposure levels, the cut-off scores proposed by Bernstein and Fink [35] for moderate and high exposure levels for the five CTQ subscales were used. For this purpose, ROC curve analysis was used to determine the discriminatory properties of the raw scores for the matching MACE subscales. ROC analysis is a statistical method used to evaluate the diagnostic performance of a binary classification test. It assesses how well the test distinguishes between two conditions – “exposure” or “non-exposure”. Diagnostic accuracy parameters (sensitivity [true positive rate – proportion of actual positives negatives that are correctly identified by the test] and specificity [true negative rate – proportion of actual negatives that are correctly identified by the test]) were extracted to determine the optimal cut-off value of the raw MACE score in the subscales. The aim was to identify as many cases as possible with a clinically relevant level of exposure, therefore sensitivity was assessed first (sensitivity of at least 0.70 was sought), followed by specificity. The optimal compromise was defined as the highest combination of sensitivity and specificity. For some subscales, it was not possible to define cut-off values adequately with the CTQ thresholds, so thresholds analogous to the Norwegian version of the MACE-55 were adopted for these scales.

Predictive validity was estimated using the Pearson correlation coefficient between MACE total scores (sum, multiplicity, sum by duration), CTQ, ACE and SCL-90 subscale scores.

All data analyses were conducted using R version 4.0.3 using the eRm package [40] and the ltm package [41], while other calculations were performed in IBM SPSS Statistics 29.0.

The following summed MACE scores were used in the statistical analysis:

(1) Ten subscale scores, summing the items with positive responses for each subscale, with each subscale score matched on a scale from 0 to 10 points.
(2) The MACE summation score (MACE SUM), which is the sum of the scores for the 10 subscales (range 0–100).
(3) Multiplicity score (MACE MULTI) for the number of different types of maltreatment (range 0–10), where each type must be reported above the clinical cut-off level calculated with reference to the clinical cut-off values in the CTQ.
(4) Total duration score (MACE Duration), which is the number of years with exposure to at least one type of maltreatment above the clinical cut-off level calculated with reference to the clinical cut-off levels in the CTQ (range 0–18).
(5) Total summation score by duration (MACE Sum by Duration), obtained by first calculating the summary score for each age level (1–18) and summing the scores for the 10 subscales in that age category, and then summing these scores for the 18 age groups and dividing the score by 18 to obtain a 0–100 range scale.

### Transparency and openness

The study sample included a clinical group (n =  101) and a general population group (n =  206), with sample size calculated according to accepted statistical standards. The study adhered to JARS guidelines [42].

Data, analytic code, and materials are publicly accessible and available from the first author upon reasonable request. This study and its analyses were not preregistered.

## Results

### Properties analysis of the Polish version of MACE

The results of the Rasch model analysis for each of the 10 subscales of MACE for the whole sample are presented first.

**Chart 1:**
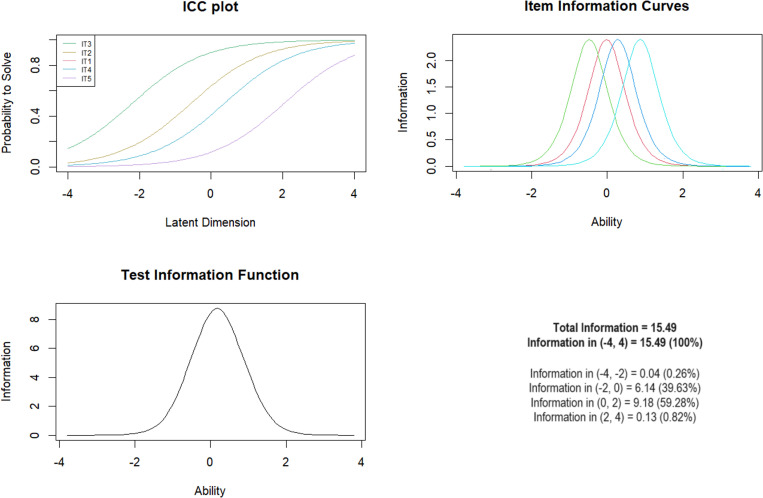
Rasch model statistics for PVA.

***Subscale 1. Parental Verbal Abuse (PVA)*** initially included 5 items and this number of items entered the final scale. Mean-square fit values ranged from 0.63 to 1.06. The Andersen test proved statistically significant when comparing median age. The Wald test showed differences for age for item IT4 (*Z* =  -3.14; *p* =  0.002). For the remaining items, equality was shown for age groups. For gender and presence of mental illness, no differences were shown in terms of model fit (Table 3). The test’s information function showed that the largest range of information was for scores in the logit value range of 0–2, indicating optimal discrimination between moderate and higher levels of exposure with 59.28% of total information (Chart 1).

**Table 3:** Item fit statistics for the PVA scale.

Items	Item difficulty β (SE)	Outfit	Infit	% Yes	Andersen LR’s test
IT1	-0.366 (0.158)	0.704	0.763	41.4%	
IT2	0.563 (0.165)	0.632	0.688	51.5%	
IT3	2.204 (0.220)	0.837	0.838	66.3%	
IT4	-0.366 (0.158)	1.056	1.045	41.4%	
IT5	-2.036 (0.190)	1.003	0.911	22.7%	
Gender as the split criterion					χ^2^(4) = 4.95; p = 0.293
Median of age as the split criterion					χ^2^(4) = 10.30; p = 0.036
Illness as the split criterion					χ^2^(4) = 8.40; p = 0.078

***Subscale 2. Emotional Neglect (EN)*** Originally, 9 MACE-X items were included in the model and this number of items remained. Item 73 was not included as in other language versions this item was included in the physical neglect subscale and this was also the case in the Polish version. The values of the mean-square fit ranged from 0.79 to 1.27. The LR test showed an adequate fit for younger and older individuals, as well as for those with and without a mental illness. Significant differences were noted by the gender of the respondents. Analysis with the Wald test showed significant gender-based differences for questions IT51 (*Z* =  2.87; *p* =  0.004), IT52 (*Z* =  3.00; *p* =  0.003), IT53 (*Z* =  1.98; *p* =  0.048) and the last two: IT74 (*Z* =  -3.52; *p* <  0.001) and IT75 (*Z* =  -3.20; *p* =  0.001) (Table 4).

**Table 4:** Item fit statistics for the EN scale.

Items	Item difficulty β (SE)	Outfit	Infit	% Yes	Andersen LR’s test
IT51	0.241 (0.135)	0.794	0.851	28.5%	
IT52	-0.612 (0.129)	1.006	1.026	43.0%	
IT53	1.353 (0.168)	0.857	0.935	13.9%	
IT54	0.495 (0.140)	1.269	1.157	24.6%	
IT56	-0.520 (0.129)	1.003	0.991	41.4%	
IT57	0.517 (0.140)	1.172	1.133	75.7%	
IT58	0.023 (0.132)	1.053	1.059	68.0%	
IT74	-0.721 (0.130)	0.835	0.850	55.0%	
IT75	-0.777 (0.130)	0.817	0.822	54.0%	
Gender as the split criterion					χ^2^(8) = 45.41; p = 0.001
Median of age as the split criterion					χ^2^(8) = 14.07; p = 0.080
Illness as the split criterion					χ^2^(8) = 3.74; p = 0.880

**Chart 2:**
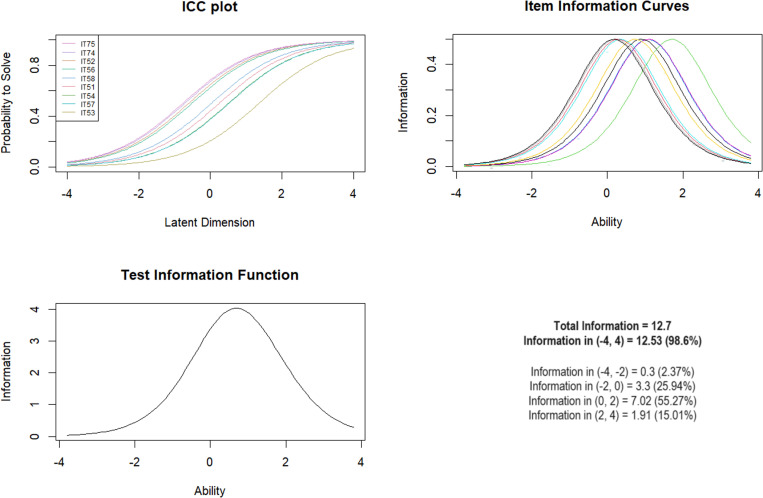
Rasch model statistics for EN.

The test information curve contained the largest interval of information in the range of logit scores 0–2 (55.27%), indicating optimal discrimination between moderate and higher levels of exposure (Chart 2).

**Chart 3:**
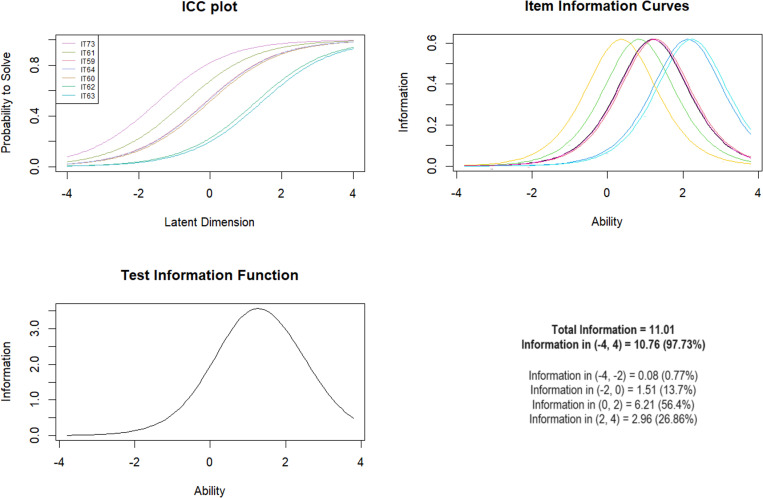
Raschad model statistics for PN.

***Subscale 3. Physical Neglect (PN)*** The PN subscale consisted of 7 items, including item 73, according to the US MACE. The mean-square fit ranged from 0.73 to 1.18. The Andersen LR test was significant when comparing the model for age groups, but not for gender and presence of mental illness. The Wald test showed significant differences between age groups for items IT61 (*Z* =  -2.97; *p* =  0.003) and IT64 (*Z* =  2.22; *p* =  0.026) – for the other items the differences were not significant (*p* >  0.05) (Table 5). The test information curve had the highest level of information in the target range 0–2 (56.4%), indicating optimal discrimination between moderate and higher exposure levels (Chart 3).

**Table 5:** Item fit statistics for the PN scale.

Items	Item difficulty β (SE)	Outfit	Infit	% Yes	Andersen LR’s test
IT59	-0.169 (0.153)	0.821	0.846	79.0%	
IT60	-0.064 (0.155)	0.776	0.830	80.3%	
IT61	-0.744 (0.146)	1.176	1.097	71.2%	
IT62	1.235 (0.207)	0.802	0.878	7.8%	
IT63	1.399 (0.218)	0.728	0.801	6.8%	
IT64	-0.143 (0.153)	1.089	1.103	20.7%	
IT73	-1.515 (0.147)	0.906	1.009	59.5%	
Gender as the split criterion					χ^2^(6) = 6.14; p = 0.407
Median of age as the split criterion					χ^2^(6) = 18.12; p = 0.006
Illness as the split criterion					χ^2^(6) = 12.54; p = 0.051

**Chart 4:**
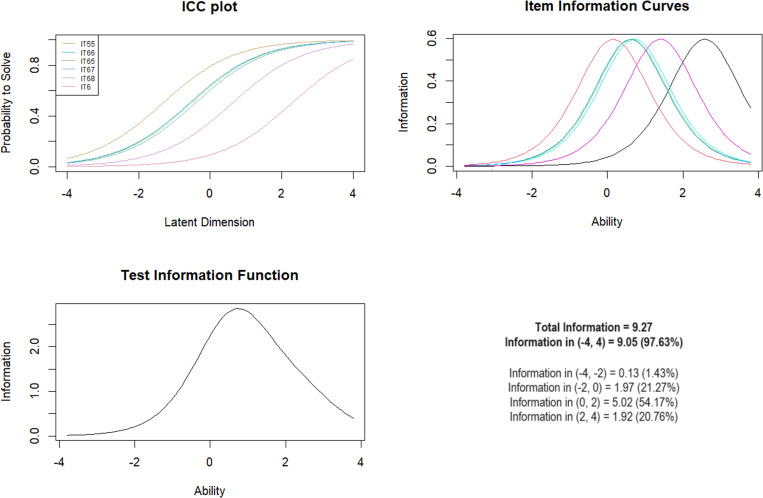
Rasch model statistics for PNVEA.

***Subscale 4. Parental Non-verbal Emotional Abuse (PNVEA)*** initially contained 6 items. All mean-square fits were within the acceptable range (0.79 to 1.10), so all 6 items remained on the scale. The overall scale did not differ from the Rasch model when split into younger and older people and by the presence of mental illness. However, differences were noted for men and women. The Wald test showed significant gender differences for items IT6 (*Z* =  -2.40; *p* =  0.017), IT65 (*Z* =  2.21; *p* =  0.027), IT67 (*Z* =  3.49; *p* <  0.001). For the other items, no differences were noted (Table 6). The largest range of information for the test information curve revealed optimal discrimination for moderate levels of exposure with 54.17% information between logit scores of 0–2 (Chart 4).

**Table 6:** Item fit statistics for the PNVEA scale.

Items	Item difficulty β (SE)	Outfit	Infit	% Yes	Andersen LR’s test
IT6	2.280 (0.249)	1.050	0.844	4.5%	
IT55	-1.330 (0.142)	1.027	1.102	46.3%	
IT65	-0.548 (0.139)	0.792	0.846	33.7%	
IT66	-0.590 (0.139)	0.830	0.883	34.3%	
IT67	-0.423 (0.140)	0.847	0.909	31.7%	
IT68	0.611 (0.156)	1.100	1.013	17.5%	
Gender as the split criterion					χ^2^(5) = 22.16; p < 0.001
Median of age as the split criterion					χ^2^(5) = 7.08; p = 0.215
Illness as the split criterion					χ^2^(5) = 7.39; p = 0.193

**Chart 5:**
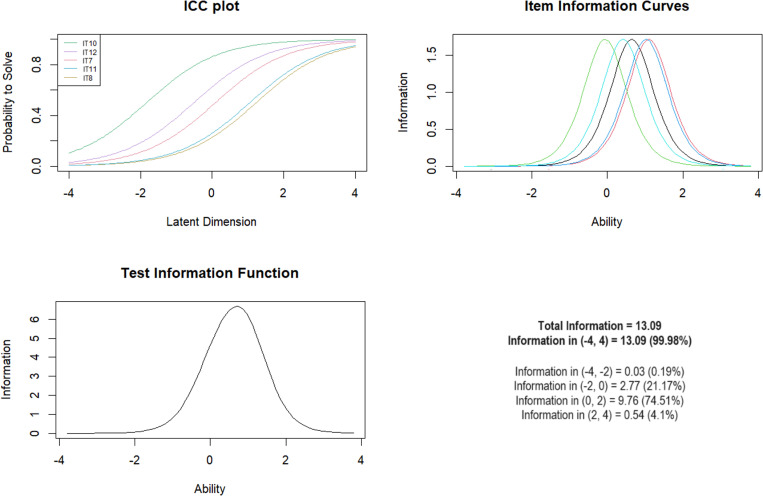
Rasch model statistics for PPA.

***Subscale 5. Parental Physical Maltreatment (PPA)*** Items 7–12 were originally considered for inclusion in the PPA scale. Item IT9 assumed a low mean-square fit =  0.32, so this item was excluded from the scale. The Andersen test showed equality of the Rasch model by gender, age and presence of mental illness (Table 7). The overall test information showed the most information between moderate levels of exposure, with 74.51% between logit scores of 0–2 (Chart 5).

**Table 7:** Item fit statistics for the PPAscale.

Items	Item difficulty β (SE)	Outfit	Infit	% Yes	Andersen LR’s test
IT7	0.086 (0.154)	0.716	0.783	29.8%	
IT8	1.225 (0.178)	0.775	0.896	18.1%	
IT10	-1.839 (0.179)	0.828	0.952	52.4%	
IT11	1.045 (0.172)	0.831	1.088	19.7%	
IT12	-0.517 (0.153)	0.750	0.827	36.9%	
Gender as the split criterion					χ^2^(4) = 2.83; p = 0.587
Median of age as the split criterion					χ^2^(4) = 2.83; p = 0.587
Illness as the split criterion					χ^2^(4) = 8.66; p = 0.070

***Subscale 6. Witnessing Violence Towards Parents (WITP)*** Eight MACE items were originally considered for inclusion (items 31–38). IT35, “Saw adults living in the household hit your mother (stepmother, grandmother) so hard, or intentionally harm her in some way, that she received or should have received medical attention.” had a low score =  0.40. This item was removed from the model, as well as the corresponding item concerning the other parent (item 38). Item 37 “Saw adults living in the household hit your father (stepfather, grandfather) so hard that it left marks for more than a few minutes.” was also excluded, as it had a low score =  0.33 and the corresponding item concerning the other parent (34). The final scale consisted of four items for which the mean-square fit was between 0.60 and 0.85.

**Chart 6:**
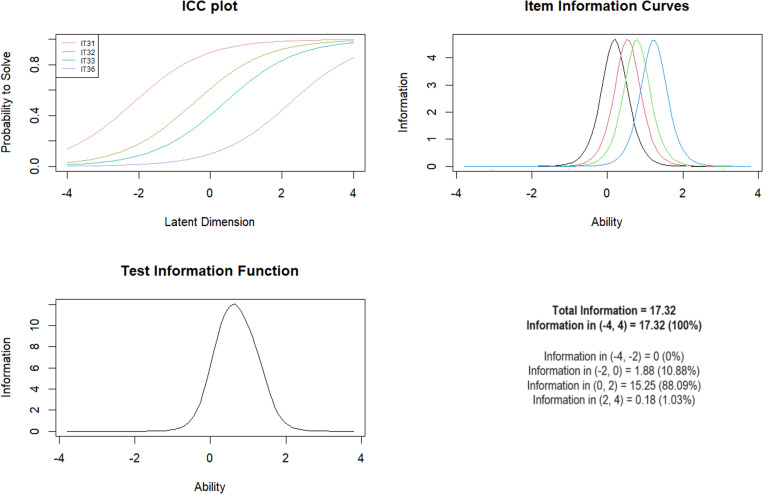
Rasch model statistics for WITP.

The Anderson test was not significant for either age, gender or the presence of mental illness as a dividing criterion (Table 8). The test information curve showed that the test was best at discriminating between moderate levels of exposure with logit scores between 0–2, including 88.09% information (Chart 6).

**Table 8:** Item fit statistics for the WITP scale.

Items	Item difficulty β (SE)	Outfit	Infit	% Yes	Andersen LR’s test
IT31	-2.158 (0.254)	0.848	0.730	46.6%	
IT32	-0.460 (0.181)	0.756	0.849	33.7%	
IT33	0.392 (0.183)	0.725	0.799	24.3%	
IT36	2.227 (0.274)	0.601	0.744	12.6%	
Gender as the split criterion					χ^2^(3) = 1.45; p = 0.693
Median of age as the split criterion					χ^2^(3) = 0.55; p = 0.907
Illness as the split criterion					χ^2^(3) = 5.08; p = 0.166

***Subscale 7. Witnessing Violence to Siblings (WITS)*** Initially, 6 MACE items were included in the model (items 18–25), with item 23 excluded as none of the subjects indicated the presence of this phenomenon. Due to a high mean square value =  2.20, IT22 was excluded and due to a low value =  0.14, IT24 was excluded. After their exclusion, all items showed values between 0.62 and 1.15. The Andersen test showed no significant differences for the overall model by gender, age and presence of mental illness (Table 9).

**Table 9:** Item fit statistics for the WITS scale.

Items	Item difficulty β (SE)	Outfit	Infit	% Yes	Andersen LR’s test
IT18	-1.550 (0.223)	0.849	0.990	20.8%	
IT19	-0.957 (0.217)	0.618	0.700	16.9%	
IT20	1.161 (0.269)	0.591	0.848	4.9%	
IT21	1.794 (0.322)	1.148	0.808	2.9%	
IT25	0.488 (0.217)	1.021	1.066	13.4%	
Gender as the split criterion					χ^2^(4) = 5.51; p = 0.239
Median of age as the split criterion					χ^2^(4) = 2.89; p = 0.576
Illness as the split criterion					χ^2^(4) = 0.91; p = 0.923

**Chart 7:**
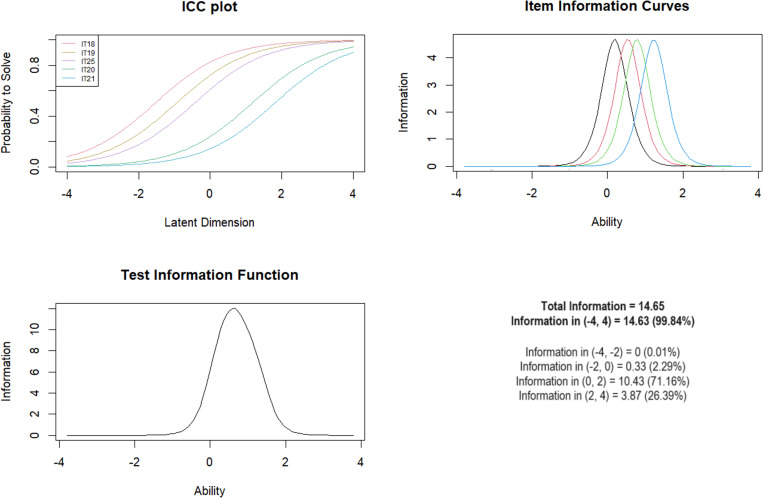
Rasch model statistics for WITS.

The peak of the test information curve showed the best discrimination for moderate exposure levels of 71.16% for logit scores between 0 and 2 (Chart 7).

***Subscale 8. Peer emotional abuse (PEERE)*** Initially, MACE items 39–43 were included in the item pool and showed an acceptable fit of 0.67 - 0.127. The Andersen test showed significant differences for the model for gender and age, but no significant differences for the presence of mental illness

**Chart 8:**
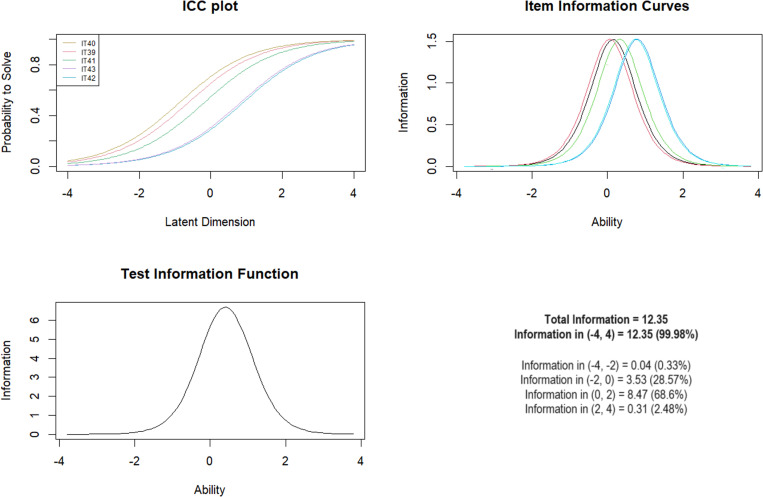
Rasch model statistics for PEERE.

Analysis with the Wald test for gender showed differences between men and women for items IT39 (*Z* =  -2.06; *p* =  0.039), IT41 (*Z* =  2.81; *p* =  0.005) and IT43 (*Z* =  -4.09; *p* <  0.001). For age, differences were noted for question IT41 (*Z* = 3.05; *p* =  0.002) and question IT43 (*Z* =  -3.59; *p* <  0.001) (Table 10). The test information function showed that the highest scale information was within the target range of 0–2, indicating optimal discrimination between moderate and higher exposure levels with 68.6% of total information between logits 0–2 (Chart 8).

**Table 10:** Item fit statistics for the PEERE scale.

Items	Item difficulty β (SE)	Outfit	Infit	% Yes	Andersen LR’s test
IT39	-0.639 (0.149)	0.926	0.945	46.0%	
IT40	-0.885 (0.153)	0.679	0.809	48.9%	
IT41	-0.192 (0.144)	0.837	0.873	40.5%	
IT42	0.896 (0.149)	0.973	0.950	26.5%	
IT43	0.819 (0.148)	1.273	1.156	27.5%	
Gender as the split criterion					χ^2^(4) = 28.70; p = 0.001
Median of age as the split criterion					χ^2^(4) = 18.23; p = 0.001
Illness as the split criterion					χ^2^(4) = 6.90; p = 0.141

**Chart 9:**
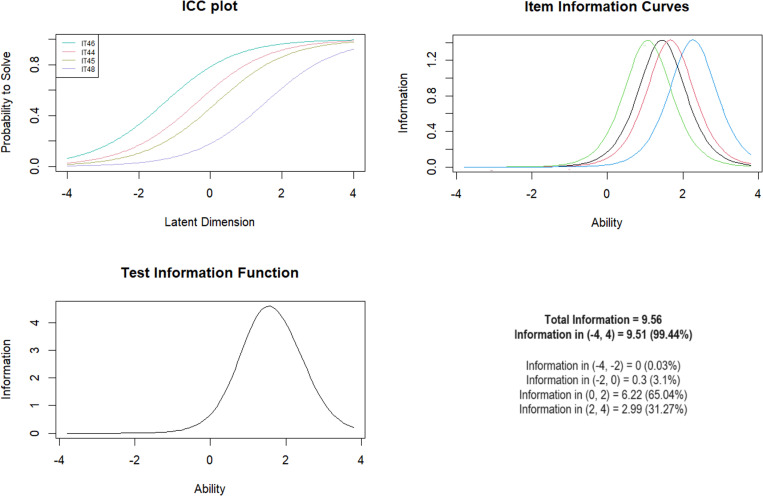
Rasch model statistics for PPB.

***Subscale 9. Peer Physical Bullying (PPB)*** Five items were included in the subscale, from 44 to 48. IT47 was removed due to a very low mean square value =  0.44. For the other items, the values after its exclusion were 0.62-1.02. The Andersen test showed no significant differences in the model for the age criterion and the presence of mental illness. However, significant differences were noted for gender. Analysis with the Wald test showed gender differences for question IT45 (*Z* =  2.60; *p* =  0.009) and IT48 (*Z* =  -1.99; *p* =  0.047) (Table 11). The test information curve showed that the test was best at discriminating moderate levels of exposure with logit scores between 0 and 2 (including 65.04% information) (Chart 9).

**Table 11:** *Item fit statistics for the* PEPB *scale.*

Items	Item difficulty β (SE)	Outfit	Infit	% Yes	Andersen LR’s test
IT44	-0.392 (0.207)	0.990	1.018	12.3%	
IT45	0.161 (0.219)	0.887	0.904	9.4%	
IT46	-1.292 (0.204)	0.735	0.857	19.4%	
IT48	1.523 (0.297)	0.617	0.787	3.6%	
Gender as the split criterion					χ^2^(3) = 12.19; p = 0.007
Median of age as the split criterion					χ^2^(3) = 3.86; p = 0.227
Illness as the split criterion					χ^2^(3) = 1.35; p = 0.716

**Chart 10:**
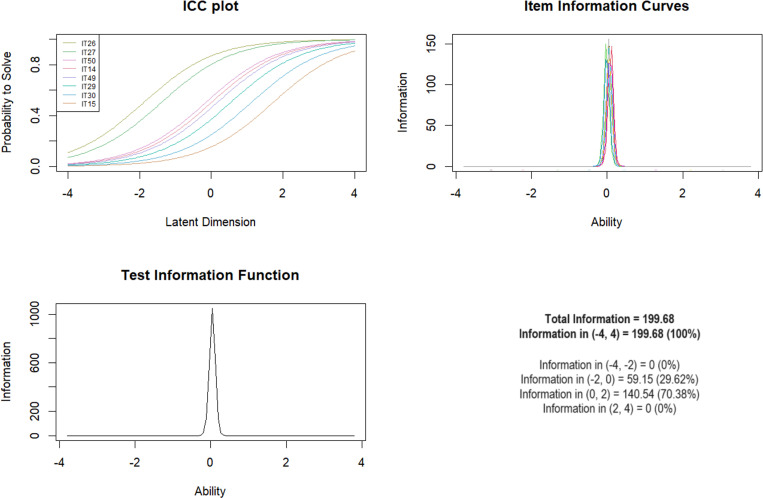
Rasch model statistics for SEXA.

***Subscale 10. Childhood Sexual Abuse (SEXA)*** Of the initial 12 items included, the final scale included 8 items. IT13 was excluded from the scale due to high Outfit values (1.73) and IT16 (0.24), IT28 (0.47) and IT17 (0.49) due to low values. The Andersen test was found to be insignificant for gender, age and presence of mental illness (Table 12). The overall test information curve revealed the most information for moderate levels of exposure with 70.38% overall information for logit scores of 0–2. It did not differentiate between subjects with extremely low and high levels of exposure (0% overall information for logit scores of -4; -2 and 2;4) (Chart 10)

**Table 12:** Item fit statistics for the SEXA scale.

Items	Item difficulty β (SE)	Outfit	Infit	% Yes	Andersen LR’s test
IT14	-0.013 (0.269)	1.057	1.090	8.7%	
IT15	1.710 (0.396)	0.791	0.806	3.9%	
IT26	-1.900 (0.248)	1.106	1.176	18.8%	
IT27	-1.405 (0.240)	0.720	0.825	15.9%	
IT29	0.524 (0.297)	0.648	0.708	6.8%	
IT30	1.101 (0.339)	0.784	1.056	5.2%	
IT49	0.152 (0.277)	0.735	0.911	8.7%	
IT50	-0.169 (0.263)	0.829	0.912	9.4%	
Gender as the split criterion					χ^2^(7) = 9.35; p = 0.228
Median of age as the split criterion					χ^2^(7) = 6.92; p = 0.437
Illness as the split criterion					χ^2^(7) = 7.10; p = 0.419

## Summary

The Polish version of MACE after analyses included a total of 58 items combined into 10 scales. Table 13 presents the items of questions matched from MACE-X to the Polish version of the tool.

**Table 13:** Characteristics of test items in the Polish version of MACE.

Subscales	Number of items	Test items from the original MACE-X scale
1. Parental verbal abuse (PVA)	5	1, 2, 3, 4, 5.
2. Emotional neglect (EN)	7	51, 52, 53, 54, 56, 57r * , 58r, 74r, 75r
3. Physical neglect (PN)	6	59r, 60r, 61, 62, 63, 64, 73r
4. Parental non-verbal emotional abuse (PNEVA)	5	6, 55, 65, 66, 67, 68
5. Parental physical maltreatment (PPA)	6	7, 8, 10, 11, 12
6. Witnessed interpersonal violence to parents (WITP)	6	31, 32, 33, 36
7. Witnessing violence to siblings (WITS)	5	18, 19, 20, 21, 25
8. Peer verbal abuse (PEERE)	4	39, 40, 41, 42, 43
9. Peer physical bullying (PPB)	4	44, 45, 46, 48
10. Childhood sexual abuse (SEXA)	8	14, 15, 26, 27, 29, 30, 49, 50.
MACE total	58	58

* Items with “r” are reversed.

### Convergent validity of MACE with CTQ and ACE

Convergent validity of MACE-58 with CTQ and ACE was estimated using Pearson correlation. Strong and positive correlations were found between MACE SUM, MACE Multiplicity and MACE Duration and the overall CTQ and ACE score (Table 14). Correlations between the CTQ subscales and the corresponding MACE subscales were also positive (Table 15), with slightly weaker correlations (moderate) reported for Physical neglect (*r* =  0.49; *p* <  0.001) and parental non-verbal emotional abuse with MACE and Emotional neglect with CTQ (*r* =  0.46; *p* <  0.001). For the other subscales, correlations were strong, as expected.

**Table 14:** Pearson correlations between MACE-58 and CTQ scores and ACE scores.

MACE		CTQ			ACE		
	M (SD)	*r*	*p*	*95% CI*	*r*	*p*	*95% CI*
MACE SUM	29.34 (19.21)	0.70	<0.001	0.64; 0.75	0,77	<0.001	0.72; 0.81
MACE Multiplicity	3.88 (2.62)	0.66	<0.001	0.59; 0.72	0.73	<0.001	0.67; 0.78
MACE Duration	11.99 (6.84)	0.52	<0.001	0.44; 0.60	0.51	<0.001	0.42; 0.59

**Table 15:** Pearson correlations between the MACE-58 subscales and the CTQ.

MACE	CTQ	*r*	*p*	*95% CI*
Parental verbal abuse	Emotional abuse	0.66	<0.001	0.59; 0.71
Emotional neglect	Emotional neglect	0.59	<0.001	0.52; 0.66
Physical neglect	Physical neglect	0.49	<0.001	0.40; 0.57
Parental non-verbal emotional abuse	Emotional abuse	0.56	<0.001	0.48; 0.63
	Emotional neglect	0.46	<0.001	0.36; 0.54
Parental physical abuse	Physical abuse	0.58	<0.001	0.50; 0.65
Sexual abuse	Sexual abuse	0.68	<0.001	0.62; 0.74

#### Predictive ability of MACE – ROC curve.

Table 16 presents the results of the ROC analysis, together with the sensitivity and specificity of predicting the presence (or not) of clinical exposure for the CTQ scales based on MACE scores. The cut-off points for MACE scores were determined mainly on the basis of sensitivity in an attempt to make its value as close to 0.7 or higher. High sensitivity for all scales means that the test is very effective in detecting all positive cases in the study population. The higher the sensitivity, the fewer cases are missed (false negatives).

**Table 16:** Predictive ability of MACE for clinical exposure of CTQ subscales.

MACE subscale	CTQ subscale	AUC [95% CI]	Best n items/cut-off*	Sensitivity/specificity
Parental verbal abuse	Emotional abuse	0.84 [0.79–0.89]	3	0.86/0.68
Emotional neglect	Emotional neglect	0.70 [0.64–0.77]	3	0.80/0.42
Physical neglect	Physical neglect	0.75 [0.69–0.81]	3	0.85/0.48
Parental non-verbal emotional abuse	Emotional abuse	0.77, [0.71–0.83]	2	0.79/0.66
	Emotional neglect	0.73 [0.67–0.79]	2	0.71/0.66
Parental physical abuse	Physical abuse	0.80 [0.73–0.87]	2	0.80/0.64
Sexual abuse	Sexual abuse	0.80 [0.72–0.87]	1	0.68/0.83

* Number of items for the MACE subscale with the highest sensitivity for the CTQ clinical cut-off points. For the four subscales with no counterpart in the CTQ, cut-off points analogous to the Norwegian version of the MACE-55 were included: Witnessing interpersonal violence to parents – 3; Witnessing violence to siblings – 2; Peer verbal/emotional abuse – 3; Peer physical bullying – 2.

### Predictive validity

Table 17 presents Pearson correlation coefficients assessing the predictive validity of MACE, CTQ, and ACE with SCL-90 subscales and the total SCL-90 score. Significant positive correlations at the moderate level were observed between MACE and CTQ scores with all SCL-90 subscales. Correlations between ACE total scores and MACE and CTQ were strong and positive, with slightly stronger correlations for MACE than for CTQ. The predictive validity of MACE was confirmed by its strong correlations with the total SCL-90 score and moderate but consistent correlations with SCL-90 subscales, indicating its capacity to predict psychopathological symptoms.

**Table 17:** Pearson correlations between MACE, CTQ and SCL-90 and ACE scores.

	MACE SUM		MACE MULTI		MACE Sum by Duration		CTQ SUM		ACE total	
	*r*	*p*	*r*	*p*	*r*	*p*	*r*	*p*	*r*	*p*
Somatization	0.39	<0.001	0.36	<0.001	0.41	<0.001	0.41	<0.001	0.31	<0.001
Obsessive compulsive	0.34	<0.001	0.31	<0.001	0.32	<0.001	0.32	<0.001	0.31	<0.001
Interpresonal sensibility	0.44	<0.001	0.42	<0.001	0.42	<0.001	0.40	<0.001	0.43	<0.001
Depression	0.42	<0.001	0.41	<0.001	0.42	<0.001	0.40	<0.001	0.41	<0.001
Anxiety	0.43	<0.001	0.41	<0.001	0.42	<0.001	0.44	<0.001	0.43	<0.001
Anger hostility	0.33	<0.001	0.30	<0.001	0.34	<0.001	0.33	<0.001	0.32	<0.001
Phobic anxiety	0.40	<0.001	0.36	<0.001	0.41	<0.001	0.40	<0.001	0.42	<0.001
Paranoid ideation	0.40	<0.001	0.40	<0.001	0.42	<0.001	0.36	<0.001	0.41	<0.001
Psychoticism	0.42	<0.001	0.41	<0.001	0.39	<0.001	0.42	<0.001	0.41	<0.001
SCL-90 total	0.47	<0.001	0.45	<0.001	0.46	<0.001	0.45	<0.001	0.44	<0.001

### Reliability

To determine the test’s reliability, Kuder-Richardson coefficients (KR20) were calculated for the individual MACE subscales, which are presented in Table 18. Most of the analyzed scales assumed a moderate level of reliability (0.5–0.8). Only two subscales PVA and SEXA had high reliability ( > 0.8) [39].

**Table 18:** Reliability coefficients for the MACE subscales.

Subscales	KR-20
1. Parental verbal abuse (PVA)	0.827
2. Emotional neglect (EN)	0.781
3. Physical neglect (PN)	0.569
4. Parental non-verbal emotional abuse (PNEVA)	0.648
5. Parental physical maltreatment (PPA)	0.782
6. Witnessed interpersonal violence to parents (WITP)	0.791
7. Witnessing violence to siblings (WITS)	0.718
8. Peer verbal abuse (PEERE)	0.795
9. Peer physical bullying (PEPB)	0.647
10. Childhood sexual abuse (SEXA)	0.867

## Discussion

The MACE is a scale commonly used around the world for retrospective assessment of abuse and neglect during development. In creating a Polish-language version of the MACE, we first translated the US version of the MACE-X [26] into polish and adapted item wordings to the sociocultural conditions of Poland. Based on data obtained from a survey of clinical groups and the general population, each of the 10 subscales in the MACE-58 (PL) consisted of 4–9 items. All scores for the scales were recoded on 0–10 scales, similar to the Norwegian and French adaptations. Age differences were observed in 3 of the 10 subscales of the MACE-58 (PL). The subscales Physical Neglect (PN) and Peer Emotional Abuse (PEA) and item IT4 (“Zachowywano się w sposób, który sprawiał, że obawiałeś/-aś się, że możesz doznać krzywdy fizycznej”) from the Parental Verbal Abuse (PVA) subscale were age-related, i.e., they were associated with a different item arrangement for older and younger participants. These results show differences among the participant group in how older respondents compared to younger respondents experienced physical neglect (or perceived neglect), peer emotional abuse and how they feared physical violence from their parents during their developmental years. However, it is imperative to draw a distinction between differential item functioning (DIF) and frequency of reporting experiences. DIF signifies disparities in the manner in which diverse groups interpret and respond to specific items, despite maintaining equivalent underlying levels of the measured construct. Conversely, frequency denotes the number of instances of an experience. It is crucial to exercise caution in interpreting both of these aspects, to avoid conflating patterns of response with actual prevalence rates. In the subscales: Emotional Neglect (EN), Parental Non-Verbal Emotional Abuse (PNVEA), Peer Emotional Abuse (PEERE) and Peer Physical Bullying (PPB) showed significant cross-gender differences, indicating different response patterns for each item for males and females. The other subscales were consistent in terms of gender and age. None of the subscales showed differences with regard to the presence of mental illness in the respondents. It is crucial to emphasize that these results do not necessarily reflect differences in the actual prevalence of experiences but may also result from DIF.

The data obtained cannot directly and unequivocally explain the reasons for the gender differences, but they are in line with what previous research on violence has indicated. However, they align with existing literature suggesting that experiences of emotional availability during childhood can have different impacts based on gender. Previous studies have indicated that women more often report their mothers’ emotional unavailability during childhood. This finding may reflect gendered socialization processes that shape emotional experiences and family dynamics. For instance, research highlights that girls might be more encouraged to form close emotional bonds within families [43], which could amplify the perceived significance of maternal emotional availability.

It is important to note that gendered responses to emotional stress are complex and not fully understood. Chaplin [44] discusses that while girls may tend to express emotions more openly and internalize negative experiences, boys often exhibit heightened physiological responses (e.g., increased cortisol levels) to emotional stress, which could manifest as externalized behaviors such as aggression or substance abuse. These differences suggest that emotional unavailability might affect men and women differently rather than disproportionately.

Furthermore, the analysis should not overlook that various adverse childhood experiences, such as sexual abuse, disproportionately affect girls and are often perpetrated by individuals other than mothers. These broader factors may also influence how emotional unavailability is perceived and reported across genders. Future research should consider such variables to provide a more nuanced understanding of how childhood experiences shape emotional development in both women and men.

The revealed gender differences in the sphere of parental non-verbal emotional abuse reflect the data we have on forms of violence applicable to women and men. For men, it was more often physical interference, such as being locked in a wardrobe or basement, while for women it involved taking over adult responsibilities, which is a form of parentification, i.e., a reversal of family roles. This is consistent with recent studies conducted on the Polish population showing that young girls are characterised by higher levels of parentification, especially emotional parentification focused on parents [45], which may be related to the cultural context and the socialisation process.

In the area of peer violence, a known pattern in the literature emerges. Women in the present study were more likely than men to report experiences of peers talking or gossiping about them during their developmental years. While this aligns with research suggesting that women, particularly adolescent women, are more likely to encounter and engage in indirect and verbal violence [46, 47], it is important to note that our data do not specify the gender of the perpetrators. Existing literature highlights that gossiping among women can sometimes function as a strategy to eliminate rivals by selectively sharing social information relevant to their reputation [48, 49]. However, societal norms and moral codes imposed disproportionately on young women may also contribute to their greater exposure to gossip and rumors. Women may be targeted for behaviors that deviate from these norms, whereas similar behaviors in men might not provoke comparable social repercussions.

It should also be noted that males in the study reported more frequent use of direct physical and verbal violence (e.g., vulgarisms, name-calling) by peers than females, a pattern consistent with previous research [47,50,51]. These findings underscore the complexity of gendered experiences with peer violence and suggest the need to interpret differences in the context of both individual behaviors and broader societal expectations.

There were mixed responses from study participants by age. Younger study participants indicated significantly more often than older participants that they had encountered behaviours in both family and peer settings that caused them to fear physical harm. They were also more likely to be gossiped about or to have abusive posts made about them on the Internet, while at the same time the home environment provided them with more help with chores. Younger respondents were more likely to indicate that they were left without adult supervision when such supervision was required. The results obtained may, on the one hand, indicate that younger persons experienced more violence in certain situations, but, on the other hand, may also indicate their greater vigilance with respect to possible harm, or their increased awareness of their own rights and needs. Perhaps the results obtained are related to the social and cultural changes that have taken place in Poland over the last decades. There are psycho-educational campaigns in the public and social media and a growing number of grassroots movements aimed at a deeper understanding of violence. The increased incidence of indirect violence using the Internet is a phenomenon that has emerged with technological development and is now widespread. State and non-governmental organisations in Poland are trying to combat it. According to research, it is younger people who are particularly exposed to violence in online environments [52] and some of these experiences relate to intimate, partner relationships [53] and online dating [54, 55]. The differences shown may also result from the fact that MACE is a retrospective questionnaire. Therefore, older people’s memories of experienced violence may not be as clear and available as the memories of younger respondents.

The data presented in this study show that the MACE-58 (PL) demonstrated good internal consistency, convergent validity with other scales assessing childhood neglect and violence, and accuracy in predicting psychopathology across all clinical symptom groups. The Polish version of the scale had high reliability, which was measured using the Kuder–Richardson formula (KR20). Only the Physical Neglect (PN) scale had a reliability of KR20 =  0.569, which suggests that caution should be exercised when interpreting the results of this scale in the future.

Convergent validity between MACE and CTQ was estimated using Pearson correlation. The analyses revealed strong to moderate correlations between the total and subscale scores of both tests, which was as expected. The convergent validity of the Polish version of MACE was also confirmed by the fact that MACE scores could predict clinically relevant exposure levels in the CTQ subscales with an acceptable level of sensitivity and specificity. Pearson correlation analysis for the relationship between MACE and CTQ and the SCL-90 and ACE dimensions showed moderate relationships between MACE and CTQ scores with all SCL-90 dimensions. Strong and positive correlations were also revealed between ACE total and MACE and CTQ. For MACE the correlations with both tests, and especially with ACE, were slightly stronger than for CTQ. The correlation of the MACE total score and the Sum by Duration MACE with the ACE total score was strong at 0.76. The association of the CTQ total score with the ACE total score was weaker (r = 0.68). The stronger correlations shown for the MACE total score, compared to the CTQ total score, with the level of most clinical symptom subscales as measured by the SCL-90, confirmed the predictive accuracy of the Polish version of MACE. These results were consistent with the results of a population-based study by Teicher and Parigger [26] conducted in the USA, where the correlations of MACE with scales indicating psychopathological symptoms were stronger than those of the CTQ and ACE tools, and also consistent with the results obtained in the Norwegian adaptation [27]. The Sum-by-Duration Score was more strongly correlated with the five subscales of the SCL-90 and identically strongly correlated in two subscales, compared to the CTQ. Although the Polish version of the MACE did not show significantly stronger correlations with the SCL-90 than the CTQ (also when analysed with the MACE time features), the level of correlation with clinical symptoms was higher than the results obtained when adapting the Norwegian version of the MACE (r ≥  0.4 in most SCL-90 subscales) [27]. In conclusion, based on the items included in the Polish version of the MACE, also taking into account their temporal parameters, it is possible to predict the occurrence of clinical symptoms and the experience of violence during the developmental period better than based on the CTQ, both in healthy individuals and in patients with mental disorders and illnesses.

### Strengths and limitations

Although mental health specialists in Poland currently have tools to assess adverse childhood experiences in their patient, there is no tools that would help assess them in a comprehensive manner. Thanks to the Polish version of MACE, clinicians will be able to psychometrically reliably determine not only the scope but also the duration of patients’ adverse experiences. Additionally, they will be able to include types of adverse experiences, such a peer violence or witnessing domestic violence, that were not included in previously available tools.

One of the tool’s strengths is its development using advanced statistical methods based on item response theory (IRT), allowing for the detection of differential item functioning (DIF) and ensuring that variations in responses reflect genuine differences rather than biases in item interpretation. Both convergent and predictive validity of the Polish version of the Scale was demonstrated. Another advantage of the present project was that the analyses were based on data obtained during surveys among persons over 18 years of age, from the general and clinical populations. Unfortunately, despite the researchers’ access to a wide range of patients treated in Day and closed Wards, only a small proportion of these patients consented to the study. Despite the researchers’ access to a wide range of patients treated in day and closed psychiatric wards, only a small proportion of these patients consented to participate in the study. Furthermore, some patients who initially agreed to complete the MACE questionnaire opted out, potentially due to the triggering nature of the traumatic memories associated with the adverse childhood experiences described in the questionnaire. This poses a potential limitation of the study, as the sample of patients who completed the questionnaire may differ in the severity of adverse childhood experiences from the broader patient population. However, it is important to reflect on findings from other contexts. For instance, in Germany, only one out of over 400 patients with severe mental illness dropped out after completing the MACE assessment [26]. This suggests that adequate training of researchers and staff in trauma-informed approaches can help mitigate the risk of dropout by providing patients with appropriate support. Integrating such practices in future studies may improve patient retention and the overall reliability of results.

Another limitation of the present study is the inability to conduct a retest to determine test-retest reliability due to the specific organisation of patients’ lives in psychiatric wards. Additionally, the wide age range of participants could affect the accuracy of retrospective self-reports, as older respondents may find it more challenging to recall childhood experiences.

## Conclusions

The present study, conducted in a clinical and population-based groups, resulted in a Polish version of the self-administered MACE for assessing maltreatment during development. This tool can be used in the work of clinicians working in mental health centres, as well as in research, to identify adverse relationship experiences during childhood and adolescence, as well as to assess the impact of these events on later psychological problems. The Polish version of the MACE contains 58 items belonging to 10 subscales and has adequate reliability as well as convergent and predictive validity.

Due to the percentage of people of different ages affected by abuse and neglect in Poland [21], MACE can be a tool that facilitates the identification and dissemination of the problem. In future research, MACE may be used to establish associations between the timing of traumatic events in childhood and adolescence and the mechanisms underlying mental disorders and illnesses. This is particularly important for clinical practice, as symptoms of trauma often co-occur with symptoms of other disorders [56] or overlap [57], and a detailed diagnostic tool that captures the chronology of a broad spectrum of patients’ experiences can aid the identification of important events related to childhood trauma or neglect. Given these issues, it may be worth conducting further research using MACE-58 (PL) to verify whether the use of the tool assists in differential diagnosis.

The wide range of assessments included in the test items of the questionnaire may broaden the diagnostic areas and contribute to a more accurate assessment and deeper understanding of the symptoms externalised by patients that may be a consequence of past traumatic experiences. The tool could be used effectively in both diagnostic and therapeutic contexts, given the range of emotional responses activated in patients from clinical groups during the adaptation stage. Taking into account the issues identified, MACE-58 (PL) will be made available under an open access license to ensure that it can be used freely in research and clinical practice.

## Supporting information

S1 File The Polish version of the MACE questionnaire. (DOCX)

S2 File The original version of the MACE questionnaire. (DOCX)

S3 File The Polish version of the MACE questionnaire translated into English. (DOCX)

S4 File The database from the Polish adaptation of the MACE questionnaire. (XLSX)

## Glossary

Adverse childhood experiences (ACE) – potentially traumatic events that occur in childhood (0–17 years). Examples include experiencing violence, abuse, or neglect, witnessing violence in the home or community, having a family member attempt or die by suicide.

Emotional neglect – defined usually as a failure to attend to the child’s emotional needs (e.g. never showing emotion while interacting with the child).

Maltreatment – the act of treating someone cruelly or violently.

Physical neglect – when a parent, guardian or custodian fails to provide for a child’s basic needs, like food, clothing, shelter, education, medical care or supervision and abandonment.

Peer abuse – is a term used to describe children abusing other children. Peer abuse can include: bullying (including online bullying and bullying because of someone’s race, religion, sexuality, disability or trans status) abuse by your girlfriend, boyfriend or partner.

Psychological abuse – also known as mental or emotional abuse, involves using verbal and non-verbal communication to try to control someone or harm them emotionally.

Physical abuse – is intentional bodily injury. Some examples include slapping, pinching, choking, kicking, shoving, or inappropriately using drugs or physical restraints.

Sexual abuse – any act of sexual contact that a person suffers, submits to, participates in, or performs as a result of force or violence, threats, fear, or deception or without having legally consented to the act.

Trauma – event or series of events that a person perceives as physically or emotionally damaging and having a long-term adverse impact on their psychological well-being and emotional and social functioning.
