# Supplementary material for: Polish adaptation of ‘Maltreatment and Abuse Chronology of Exposure’ scale
Source: PLoS One. 2025 Apr 9;20(4):e0321046. doi: 10.1371/journal.pone.0321046 (PMC11981210; doi:10.1371/journal.pone.0321046)
Supplement: S1 File — (DOCX) [file pone.0321046.s001.docx]

MACE-58

|  | **Sometimes parents, stepparents or other adults living in the house do hurtful things.**  **If this happened during your childhood (first 18 years of your life) please provide your best estimate of your age at the time(s) of occurrence.**  **Please check all ages that apply.** | |  |  | |  |
| --- | --- | --- | --- | --- | --- | --- |
|  | | *For example item 1.* Swore at you, called you names, said insulting things like your “fat”, “ugly”, “stupid”, etc. more than a few times a year.  *If at ages 6-8 your father swore at you and at ages 8-10 your mother insulted you, and at age 17 your mother’s new live-in boyfriend called you names; you would check off as follows:*   \| 1 \| 2 \| 3 \| 4 \| 5 \| 6 \| 7 \| 8 \| 9 \| 10 \| 11 \| 12 \| 13 \| 14 \| 15 \| 16 \| 17 \| 18 \| \| --- \| --- \| --- \| --- \| --- \| --- \| --- \| --- \| --- \| --- \| --- \| --- \| --- \| --- \| --- \| --- \| --- \| --- \| \|  \|  \|  \|  \|  \| ✓ \| ✓ \| ✓ \| ✓ \| ✓ \|  \|  \|  \|  \|  \|  \| ✓ \|  \| | ⚫  Yes | 🔿  No |  | |
| 1. | | Swore at you, called you names, said insulting things like your “fat”, “ugly”, “stupid”, etc. more than a few times a year.  Please check all ages that apply.   \| 1 \| 2 \| 3 \| 4 \| 5 \| 6 \| 7 \| 8 \| 9 \| 10 \| 11 \| 12 \| 13 \| 14 \| 15 \| 16 \| 17 \| 18 \| \| --- \| --- \| --- \| --- \| --- \| --- \| --- \| --- \| --- \| --- \| --- \| --- \| --- \| --- \| --- \| --- \| --- \| --- \| \|  \|  \|  \|  \|  \|  \|  \|  \|  \|  \|  \|  \|  \|  \|  \|  \|  \|  \| | 🔿  Yes_1_ | 🔿  No_0_ |  | |
| 2. | | Said hurtful things that made you feel bad, embarrassed or humiliated more than a few times a year.  Please check all ages that apply.   \| 1 \| 2 \| 3 \| 4 \| 5 \| 6 \| 7 \| 8 \| 9 \| 10 \| 11 \| 12 \| 13 \| 14 \| 15 \| 16 \| 17 \| 18 \| \| --- \| --- \| --- \| --- \| --- \| --- \| --- \| --- \| --- \| --- \| --- \| --- \| --- \| --- \| --- \| --- \| --- \| --- \| \|  \|  \|  \|  \|  \|  \|  \|  \|  \|  \|  \|  \|  \|  \|  \|  \|  \|  \| | 🔿  Yes_1_ | 🔿  No_0_ |  | |
| 3. | | Yelled or screamed at you more than a few times per year.  Please check all ages that apply.   \| 1 \| 2 \| 3 \| 4 \| 5 \| 6 \| 7 \| 8 \| 9 \| 10 \| 11 \| 12 \| 13 \| 14 \| 15 \| 16 \| 17 \| 18 \| \| --- \| --- \| --- \| --- \| --- \| --- \| --- \| --- \| --- \| --- \| --- \| --- \| --- \| --- \| --- \| --- \| --- \| --- \| \|  \|  \|  \|  \|  \|  \|  \|  \|  \|  \|  \|  \|  \|  \|  \|  \|  \|  \| | 🔿  Yes_1_ | 🔿  No_0_ |  | |
| 4. | | Acted in a way that made you afraid that you might be physically hurt.  Please check all ages that apply.   \| 1 \| 2 \| 3 \| 4 \| 5 \| 6 \| 7 \| 8 \| 9 \| 10 \| 11 \| 12 \| 13 \| 14 \| 15 \| 16 \| 17 \| 18 \| \| --- \| --- \| --- \| --- \| --- \| --- \| --- \| --- \| --- \| --- \| --- \| --- \| --- \| --- \| --- \| --- \| --- \| --- \| \|  \|  \|  \|  \|  \|  \|  \|  \|  \|  \|  \|  \|  \|  \|  \|  \|  \|  \|   Please indicate if this made you feel helpless or terrified. | 🔿  Yes_1_  ❒  Helpless | 🔿  No_0_  ❒  Terrified |  | |
| 5. | | Threatened to leave or abandon you.  Please check all ages that apply.   \| 1 \| 2 \| 3 \| 4 \| 5 \| 6 \| 7 \| 8 \| 9 \| 10 \| 11 \| 12 \| 13 \| 14 \| 15 \| 16 \| 17 \| 18 \| \| --- \| --- \| --- \| --- \| --- \| --- \| --- \| --- \| --- \| --- \| --- \| --- \| --- \| --- \| --- \| --- \| --- \| --- \| \|  \|  \|  \|  \|  \|  \|  \|  \|  \|  \|  \|  \|  \|  \|  \|  \|  \|  \|   Please indicate if this made you feel helpless or terrified. | 🔿  Yes_1_  ❒  Helpless | 🔿  No_0_  ❒  Terrified |  | |
| 6. | | Locked you in a closet, attic, basement or garage.  Please check all ages that apply.   \| 1 \| 2 \| 3 \| 4 \| 5 \| 6 \| 7 \| 8 \| 9 \| 10 \| 11 \| 12 \| 13 \| 14 \| 15 \| 16 \| 17 \| 18 \| \| --- \| --- \| --- \| --- \| --- \| --- \| --- \| --- \| --- \| --- \| --- \| --- \| --- \| --- \| --- \| --- \| --- \| --- \| \|  \|  \|  \|  \|  \|  \|  \|  \|  \|  \|  \|  \|  \|  \|  \|  \|  \|  \|   Please indicate if this made you feel helpless or terrified. | 🔿  Yes_1_  ❒  Helpless | 🔿  No_0_  ❒  Terrified |  | |

| 7. | Intentionally pushed, grabbed, shoved, slapped, pinched, punched or kicked you.  Please check all ages that apply.   \| 1 \| 2 \| 3 \| 4 \| 5 \| 6 \| 7 \| 8 \| 9 \| 10 \| 11 \| 12 \| 13 \| 14 \| 15 \| 16 \| 17 \| 18 \| \| --- \| --- \| --- \| --- \| --- \| --- \| --- \| --- \| --- \| --- \| --- \| --- \| --- \| --- \| --- \| --- \| --- \| --- \| \|  \|  \|  \|  \|  \|  \|  \|  \|  \|  \|  \|  \|  \|  \|  \|  \|  \|  \|   Please indicate if this made you feel helpless or terrified. | 🔿  Yes_1_  ❒  Helpless | 🔿  No_0_  ❒  Terrified |  |
| --- | --- | --- | --- | --- | --- | --- | --- | --- | --- | --- | --- | --- | --- | --- | --- | --- | --- | --- | --- | --- | --- | --- | --- | --- | --- | --- | --- | --- | --- | --- | --- | --- | --- | --- | --- | --- | --- | --- | --- | --- |
| 8. | Hit you so hard that it left marks for more than a few minutes.  Please check all ages that apply.   \| 1 \| 2 \| 3 \| 4 \| 5 \| 6 \| 7 \| 8 \| 9 \| 10 \| 11 \| 12 \| 13 \| 14 \| 15 \| 16 \| 17 \| 18 \| \| --- \| --- \| --- \| --- \| --- \| --- \| --- \| --- \| --- \| --- \| --- \| --- \| --- \| --- \| --- \| --- \| --- \| --- \| \|  \|  \|  \|  \|  \|  \|  \|  \|  \|  \|  \|  \|  \|  \|  \|  \|  \|  \|   Please indicate if this made you feel helpless or terrified. | 🔿  Yes_1_  ❒  Helpless | 🔿  No_0_  ❒  Terrified |  |
| 9. | Spanked you on your buttocks, arms or legs.  Please check all ages that apply.   \| 1 \| 2 \| 3 \| 4 \| 5 \| 6 \| 7 \| 8 \| 9 \| 10 \| 11 \| 12 \| 13 \| 14 \| 15 \| 16 \| 17 \| 18 \| \| --- \| --- \| --- \| --- \| --- \| --- \| --- \| --- \| --- \| --- \| --- \| --- \| --- \| --- \| --- \| --- \| --- \| --- \| \|  \|  \|  \|  \|  \|  \|  \|  \|  \|  \|  \|  \|  \|  \|  \|  \|  \|  \|   Please indicate if this made you feel helpless or terrified. | 🔿  Yes_1_  ❒  Helpless | 🔿  No_0_  ❒  Terrified |  |
| 10. | Spanked you on your bare (unclothed) buttocks.  Please check all ages that apply.   \| 1 \| 2 \| 3 \| 4 \| 5 \| 6 \| 7 \| 8 \| 9 \| 10 \| 11 \| 12 \| 13 \| 14 \| 15 \| 16 \| 17 \| 18 \| \| --- \| --- \| --- \| --- \| --- \| --- \| --- \| --- \| --- \| --- \| --- \| --- \| --- \| --- \| --- \| --- \| --- \| --- \| \|  \|  \|  \|  \|  \|  \|  \|  \|  \|  \|  \|  \|  \|  \|  \|  \|  \|  \|   Please indicate if this made you feel helpless or terrified. | 🔿  Yes_1_  ❒  Helpless | 🔿  No_0_  ❒  Terrified |  |
| 11. | Spanked you with an object such as a strap, belt, brush, paddle, rod, etc.  Please check all ages that apply.   \| 1 \| 2 \| 3 \| 4 \| 5 \| 6 \| 7 \| 8 \| 9 \| 10 \| 11 \| 12 \| 13 \| 14 \| 15 \| 16 \| 17 \| 18 \| \| --- \| --- \| --- \| --- \| --- \| --- \| --- \| --- \| --- \| --- \| --- \| --- \| --- \| --- \| --- \| --- \| --- \| --- \| \|  \|  \|  \|  \|  \|  \|  \|  \|  \|  \|  \|  \|  \|  \|  \|  \|  \|  \|   Please indicate if this made you feel helpless or terrified. | 🔿  Yes_1_  ❒  Helpless | 🔿  No_0_  ❒  Terrified |  |

| 12. | Touched or fondled your body in a sexual way.  Please check all ages that apply.   \| 1 \| 2 \| 3 \| 4 \| 5 \| 6 \| 7 \| 8 \| 9 \| 10 \| 11 \| 12 \| 13 \| 14 \| 15 \| 16 \| 17 \| 18 \| \| --- \| --- \| --- \| --- \| --- \| --- \| --- \| --- \| --- \| --- \| --- \| --- \| --- \| --- \| --- \| --- \| --- \| --- \| \|  \|  \|  \|  \|  \|  \|  \|  \|  \|  \|  \|  \|  \|  \|  \|  \|  \|  \|   Please indicate if this made you feel helpless or terrified. | 🔿  Yes_1_  ❒  Helpless | 🔿  No_0_  ❒  Terrified |  |
| --- | --- | --- | --- | --- | --- | --- | --- | --- | --- | --- | --- | --- | --- | --- | --- | --- | --- | --- | --- | --- | --- | --- | --- | --- | --- | --- | --- | --- | --- | --- | --- | --- | --- | --- | --- | --- | --- | --- | --- | --- |
| 13. | Had you touch their body in a sexual way.  Please check all ages that apply.   \| 1 \| 2 \| 3 \| 4 \| 5 \| 6 \| 7 \| 8 \| 9 \| 10 \| 11 \| 12 \| 13 \| 14 \| 15 \| 16 \| 17 \| 18 \| \| --- \| --- \| --- \| --- \| --- \| --- \| --- \| --- \| --- \| --- \| --- \| --- \| --- \| --- \| --- \| --- \| --- \| --- \| \|  \|  \|  \|  \|  \|  \|  \|  \|  \|  \|  \|  \|  \|  \|  \|  \|  \|  \|   Please indicate if this made you feel helpless or terrified. | 🔿  Yes_1_  ❒  Helpless | 🔿  No_0_  ❒  Terrified |  |

|  | **Sometimes parents, stepparents or other adults living in the house do hurtful things to your siblings (brother, sister, stepsiblings).**  **If this happened during your childhood (first 18 years of your life) please provide your best estimates of your age at the time(s) of occurrence.**  **Please check all ages that apply.** | | |  | |  | |
| --- | --- | --- | --- | --- | --- | --- | --- |
| 14. | | Intentionally pushed, grabbed, shoved, slapped, pinched, punched, or kicked your sibling (stepsibling).  Please check all ages that apply.   \| 1 \| 2 \| 3 \| 4 \| 5 \| 6 \| 7 \| 8 \| 9 \| 10 \| 11 \| 12 \| 13 \| 14 \| 15 \| 16 \| 17 \| 18 \| \| --- \| --- \| --- \| --- \| --- \| --- \| --- \| --- \| --- \| --- \| --- \| --- \| --- \| --- \| --- \| --- \| --- \| --- \| \|  \|  \|  \|  \|  \|  \|  \|  \|  \|  \|  \|  \|  \|  \|  \|  \|  \|  \|   Please indicate if this made you feel helpless or terrified. | 🔿  Yes_1_  ❒  Helpless | | 🔿  No_0_  ❒  Terrified | |  |
| 15. | | Hit your sibling (stepsibling) so hard that it left marks for more than a few minutes.  Please check all ages that apply.   \| 1 \| 2 \| 3 \| 4 \| 5 \| 6 \| 7 \| 8 \| 9 \| 10 \| 11 \| 12 \| 13 \| 14 \| 15 \| 16 \| 17 \| 18 \| \| --- \| --- \| --- \| --- \| --- \| --- \| --- \| --- \| --- \| --- \| --- \| --- \| --- \| --- \| --- \| --- \| --- \| --- \| \|  \|  \|  \|  \|  \|  \|  \|  \|  \|  \|  \|  \|  \|  \|  \|  \|  \|  \|   Please indicate if this made you feel helpless or terrified. | 🔿  Yes_1_  ❒  Helpless | | 🔿  No_0_  ❒  Terrified | |  |

| 16. | Hit your sibling (stepsibling) so hard, or intentionally harmed him/her in some way, that he/she received or should have received medical attention.  Please check all ages that apply.   \| 1 \| 2 \| 3 \| 4 \| 5 \| 6 \| 7 \| 8 \| 9 \| 10 \| 11 \| 12 \| 13 \| 14 \| 15 \| 16 \| 17 \| 18 \| \| --- \| --- \| --- \| --- \| --- \| --- \| --- \| --- \| --- \| --- \| --- \| --- \| --- \| --- \| --- \| --- \| --- \| --- \| \|  \|  \|  \|  \|  \|  \|  \|  \|  \|  \|  \|  \|  \|  \|  \|  \|  \|  \|   Please indicate if this made you feel helpless or terrified. | 🔿  Yes_1_  ❒  Helpless | 🔿  No_0_  ❒  Terrified |  |
| --- | --- | --- | --- | --- | --- | --- | --- | --- | --- | --- | --- | --- | --- | --- | --- | --- | --- | --- | --- | --- | --- | --- | --- | --- | --- | --- | --- | --- | --- | --- | --- | --- | --- | --- | --- | --- | --- | --- | --- | --- |
| 17. | Made inappropriate sexual comments or suggestions to your sibling (stepsibling).  Please check all ages that apply.   \| 1 \| 2 \| 3 \| 4 \| 5 \| 6 \| 7 \| 8 \| 9 \| 10 \| 11 \| 12 \| 13 \| 14 \| 15 \| 16 \| 17 \| 18 \| \| --- \| --- \| --- \| --- \| --- \| --- \| --- \| --- \| --- \| --- \| --- \| --- \| --- \| --- \| --- \| --- \| --- \| --- \| \|  \|  \|  \|  \|  \|  \|  \|  \|  \|  \|  \|  \|  \|  \|  \|  \|  \|  \|   Please indicate if this made you feel helpless or terrified. | 🔿  Yes_1_  ❒  Helpless | 🔿  No_0_  ❒  Terrified |  |
| 18. | Threatened to harm your sibling (stepsibling).  Please check all ages that apply.   \| 1 \| 2 \| 3 \| 4 \| 5 \| 6 \| 7 \| 8 \| 9 \| 10 \| 11 \| 12 \| 13 \| 14 \| 15 \| 16 \| 17 \| 18 \| \| --- \| --- \| --- \| --- \| --- \| --- \| --- \| --- \| --- \| --- \| --- \| --- \| --- \| --- \| --- \| --- \| --- \| --- \| \|  \|  \|  \|  \|  \|  \|  \|  \|  \|  \|  \|  \|  \|  \|  \|  \|  \|  \|   Please indicate if this made you feel helpless or terrified. | 🔿  Yes_1_  ❒  Helpless | 🔿  No_0_  ❒  Terrified |  |

|  | **Sometimes adults or older individuals NOT living in the house do hurtful things to you.**  **If this happened during your childhood (first 18 years of your life) please provide your best estimates of your age at the time(s) of occurrence.**  **Please check all ages that apply.** | | |  | |  | |
| --- | --- | --- | --- | --- | --- | --- | --- |
| 19. | | Made inappropriate sexual comments or suggestions to you.  Please check all ages that apply.   \| 1 \| 2 \| 3 \| 4 \| 5 \| 6 \| 7 \| 8 \| 9 \| 10 \| 11 \| 12 \| 13 \| 14 \| 15 \| 16 \| 17 \| 18 \| \| --- \| --- \| --- \| --- \| --- \| --- \| --- \| --- \| --- \| --- \| --- \| --- \| --- \| --- \| --- \| --- \| --- \| --- \| \|  \|  \|  \|  \|  \|  \|  \|  \|  \|  \|  \|  \|  \|  \|  \|  \|  \|  \|   Please indicate if this made you feel helpless or terrified. | 🔿  Yes_1_  ❒  Helpless | | 🔿  No_0_  ❒  Terrified | |  |
| 20. | | Touched or fondled your body in a sexual way.  Please check all ages that apply.   \| 1 \| 2 \| 3 \| 4 \| 5 \| 6 \| 7 \| 8 \| 9 \| 10 \| 11 \| 12 \| 13 \| 14 \| 15 \| 16 \| 17 \| 18 \| \| --- \| --- \| --- \| --- \| --- \| --- \| --- \| --- \| --- \| --- \| --- \| --- \| --- \| --- \| --- \| --- \| --- \| --- \| \|  \|  \|  \|  \|  \|  \|  \|  \|  \|  \|  \|  \|  \|  \|  \|  \|  \|  \|   Please indicate if this made you feel helpless or terrified. | 🔿  Yes_1_  ❒  Helpless | | 🔿  No_0_  ❒  Terrified | |  |
| 21. | | Attempted to have any type of sexual intercourse (oral, anal or vaginal) with you.  Please check all ages that apply.   \| 1 \| 2 \| 3 \| 4 \| 5 \| 6 \| 7 \| 8 \| 9 \| 10 \| 11 \| 12 \| 13 \| 14 \| 15 \| 16 \| 17 \| 18 \| \| --- \| --- \| --- \| --- \| --- \| --- \| --- \| --- \| --- \| --- \| --- \| --- \| --- \| --- \| --- \| --- \| --- \| --- \| \|  \|  \|  \|  \|  \|  \|  \|  \|  \|  \|  \|  \|  \|  \|  \|  \|  \|  \|   Please indicate if this made you feel helpless or terrified. | 🔿  Yes_1_  ❒  Helpless | | 🔿  No_0_  ❒  Terrified | |  |
| 22. | | Actually had sexual intercourse (oral, anal or vaginal) with you.  Please check all ages that apply.   \| 1 \| 2 \| 3 \| 4 \| 5 \| 6 \| 7 \| 8 \| 9 \| 10 \| 11 \| 12 \| 13 \| 14 \| 15 \| 16 \| 17 \| 18 \| \| --- \| --- \| --- \| --- \| --- \| --- \| --- \| --- \| --- \| --- \| --- \| --- \| --- \| --- \| --- \| --- \| --- \| --- \| \|  \|  \|  \|  \|  \|  \|  \|  \|  \|  \|  \|  \|  \|  \|  \|  \|  \|  \|   Please indicate if this made you feel helpless or terrified. | 🔿  Yes_1_  ❒  Helpless | | 🔿  No_0_  ❒  Terrified | |  |

|  | **Sometimes intense arguments or physical fights occur between parents, stepparents or other adults (boyfriends, girlfriends, grandparents) living in the household.**  **If this happened during your childhood (first 18 years of your life) please provide your best estimates of your age at the time(s) of occurrence.**  **Please check all ages that apply.** | | |  | |  | |
| --- | --- | --- | --- | --- | --- | --- | --- |
| 23. | | Witnessed adults living in the household argue intensely with your mother (stepmother, grandmother), say derogatory things to her, or threaten her with harm.  Please check all ages that apply.   \| 1 \| 2 \| 3 \| 4 \| 5 \| 6 \| 7 \| 8 \| 9 \| 10 \| 11 \| 12 \| 13 \| 14 \| 15 \| 16 \| 17 \| 18 \| \| --- \| --- \| --- \| --- \| --- \| --- \| --- \| --- \| --- \| --- \| --- \| --- \| --- \| --- \| --- \| --- \| --- \| --- \| \|  \|  \|  \|  \|  \|  \|  \|  \|  \|  \|  \|  \|  \|  \|  \|  \|  \|  \|   Please indicate if this made you feel helpless or terrified. | 🔿  Yes_1_  ❒  Helpless | | 🔿  No_0_  ❒  Terrified | |  |
| 24. | | Witnessed adults living in the household argue intensely with your father (stepfather, grandfather), say derogatory things to him, or threaten him with harm.  Please check all ages that apply.   \| 1 \| 2 \| 3 \| 4 \| 5 \| 6 \| 7 \| 8 \| 9 \| 10 \| 11 \| 12 \| 13 \| 14 \| 15 \| 16 \| 17 \| 18 \| \| --- \| --- \| --- \| --- \| --- \| --- \| --- \| --- \| --- \| --- \| --- \| --- \| --- \| --- \| --- \| --- \| --- \| --- \| \|  \|  \|  \|  \|  \|  \|  \|  \|  \|  \|  \|  \|  \|  \|  \|  \|  \|  \|   Please indicate if this made you feel helpless or terrified. | 🔿  Yes_1_  ❒  Helpless | | 🔿  No_0_  ❒  Terrified | |  |
| 25. | | Saw adults living in the household push, grab, slap or throw something at your mother (stepmother, grandmother).  Please check all ages that apply.   \| 1 \| 2 \| 3 \| 4 \| 5 \| 6 \| 7 \| 8 \| 9 \| 10 \| 11 \| 12 \| 13 \| 14 \| 15 \| 16 \| 17 \| 18 \| \| --- \| --- \| --- \| --- \| --- \| --- \| --- \| --- \| --- \| --- \| --- \| --- \| --- \| --- \| --- \| --- \| --- \| --- \| \|  \|  \|  \|  \|  \|  \|  \|  \|  \|  \|  \|  \|  \|  \|  \|  \|  \|  \|   Please indicate if this made you feel helpless or terrified. | 🔿  Yes_1_  ❒  Helpless | | 🔿  No_0_  ❒  Terrified | |  |
| 26. | | Saw adults living in the household push, grab, slap or throw something at your father (stepfather, grandfather).  Please check all ages that apply.   \| 1 \| 2 \| 3 \| 4 \| 5 \| 6 \| 7 \| 8 \| 9 \| 10 \| 11 \| 12 \| 13 \| 14 \| 15 \| 16 \| 17 \| 18 \| \| --- \| --- \| --- \| --- \| --- \| --- \| --- \| --- \| --- \| --- \| --- \| --- \| --- \| --- \| --- \| --- \| --- \| --- \| \|  \|  \|  \|  \|  \|  \|  \|  \|  \|  \|  \|  \|  \|  \|  \|  \|  \|  \|   Please indicate if this made you feel helpless or terrified. | 🔿  Yes_1_  ❒  Helpless | | 🔿  No_0_  ❒  Terrified | |  |

|  | **Sometimes children your own age or older do hurtful things like bully or harass you.**  **If this happened during your childhood (first 18 years of your life) please provide your best estimates of your age at the time(s) of occurrence.**  **Please check all ages that apply.** | | |  | |  | |
| --- | --- | --- | --- | --- | --- | --- | --- |
| 27. | | Swore at you, called you names, said insulting things like your “fat”, “ugly”, “stupid”, etc. more than a few times a year.  Please check all ages that apply.   \| 1 \| 2 \| 3 \| 4 \| 5 \| 6 \| 7 \| 8 \| 9 \| 10 \| 11 \| 12 \| 13 \| 14 \| 15 \| 16 \| 17 \| 18 \| \| --- \| --- \| --- \| --- \| --- \| --- \| --- \| --- \| --- \| --- \| --- \| --- \| --- \| --- \| --- \| --- \| --- \| --- \| \|  \|  \|  \|  \|  \|  \|  \|  \|  \|  \|  \|  \|  \|  \|  \|  \|  \|  \|   Please indicate ages when (if) the person doing this to you was a date (e.g., boyfriend, girlfriend, someone you associated with on a social, romantic or intimate level).   \| 1 \| 2 \| 3 \| 4 \| 5 \| 6 \| 7 \| 8 \| 9 \| 10 \| 11 \| 12 \| 13 \| 14 \| 15 \| 16 \| 17 \| 18 \| \| --- \| --- \| --- \| --- \| --- \| --- \| --- \| --- \| --- \| --- \| --- \| --- \| --- \| --- \| --- \| --- \| --- \| --- \| \|  \|  \|  \|  \|  \|  \|  \|  \|  \|  \|  \|  \|  \|  \|  \|  \|  \|  \| | 🔿  Yes_1_  🔿  Yes_1_ | | 🔿  No_0_  🔿  No_0_ | |  |
| 28. | | Said hurtful things that made you feel bad, embarrassed or humiliated more than a few times a year.  Please check all ages that apply.   \| 1 \| 2 \| 3 \| 4 \| 5 \| 6 \| 7 \| 8 \| 9 \| 10 \| 11 \| 12 \| 13 \| 14 \| 15 \| 16 \| 17 \| 18 \| \| --- \| --- \| --- \| --- \| --- \| --- \| --- \| --- \| --- \| --- \| --- \| --- \| --- \| --- \| --- \| --- \| --- \| --- \| \|  \|  \|  \|  \|  \|  \|  \|  \|  \|  \|  \|  \|  \|  \|  \|  \|  \|  \|   Please indicate ages when (if) the person doing this to you was a date.   \| 1 \| 2 \| 3 \| 4 \| 5 \| 6 \| 7 \| 8 \| 9 \| 10 \| 11 \| 12 \| 13 \| 14 \| 15 \| 16 \| 17 \| 18 \| \| --- \| --- \| --- \| --- \| --- \| --- \| --- \| --- \| --- \| --- \| --- \| --- \| --- \| --- \| --- \| --- \| --- \| --- \| \|  \|  \|  \|  \|  \|  \|  \|  \|  \|  \|  \|  \|  \|  \|  \|  \|  \|  \| | 🔿  Yes_1_  🔿  Yes_1_ | | 🔿  No_0_  🔿  No_0_ | |  |

| 29. | Said things behind your back, posted derogatory messages about you, or spread rumors about you.  Please check all ages that apply.   \| 1 \| 2 \| 3 \| 4 \| 5 \| 6 \| 7 \| 8 \| 9 \| 10 \| 11 \| 12 \| 13 \| 14 \| 15 \| 16 \| 17 \| 18 \| \| --- \| --- \| --- \| --- \| --- \| --- \| --- \| --- \| --- \| --- \| --- \| --- \| --- \| --- \| --- \| --- \| --- \| --- \| \|  \|  \|  \|  \|  \|  \|  \|  \|  \|  \|  \|  \|  \|  \|  \|  \|  \|  \|   Please indicate ages when (if) the person doing this to you was a date.   \| 1 \| 2 \| 3 \| 4 \| 5 \| 6 \| 7 \| 8 \| 9 \| 10 \| 11 \| 12 \| 13 \| 14 \| 15 \| 16 \| 17 \| 18 \| \| --- \| --- \| --- \| --- \| --- \| --- \| --- \| --- \| --- \| --- \| --- \| --- \| --- \| --- \| --- \| --- \| --- \| --- \| \|  \|  \|  \|  \|  \|  \|  \|  \|  \|  \|  \|  \|  \|  \|  \|  \|  \|  \| | 🔿  Yes_1_  🔿  Yes_1_ | 🔿  No_0_  🔿  No_0_ |  |
| --- | --- | --- | --- | --- | --- | --- | --- | --- | --- | --- | --- | --- | --- | --- | --- | --- | --- | --- | --- | --- | --- | --- | --- | --- | --- | --- | --- | --- | --- | --- | --- | --- | --- | --- | --- | --- | --- | --- | --- | --- | --- | --- | --- | --- | --- | --- | --- | --- | --- | --- | --- | --- | --- | --- | --- | --- | --- | --- | --- | --- | --- | --- | --- | --- | --- | --- | --- | --- | --- | --- | --- | --- | --- | --- | --- | --- |
| 30. | Intentionally excluded you from activities or groups.  Please check all ages that apply.   \| 1 \| 2 \| 3 \| 4 \| 5 \| 6 \| 7 \| 8 \| 9 \| 10 \| 11 \| 12 \| 13 \| 14 \| 15 \| 16 \| 17 \| 18 \| \| --- \| --- \| --- \| --- \| --- \| --- \| --- \| --- \| --- \| --- \| --- \| --- \| --- \| --- \| --- \| --- \| --- \| --- \| \|  \|  \|  \|  \|  \|  \|  \|  \|  \|  \|  \|  \|  \|  \|  \|  \|  \|  \|   Please indicate ages when (if) the person doing this to you was a date.   \| 1 \| 2 \| 3 \| 4 \| 5 \| 6 \| 7 \| 8 \| 9 \| 10 \| 11 \| 12 \| 13 \| 14 \| 15 \| 16 \| 17 \| 18 \| \| --- \| --- \| --- \| --- \| --- \| --- \| --- \| --- \| --- \| --- \| --- \| --- \| --- \| --- \| --- \| --- \| --- \| --- \| \|  \|  \|  \|  \|  \|  \|  \|  \|  \|  \|  \|  \|  \|  \|  \|  \|  \|  \| | 🔿  Yes_1_  🔿  Yes_1_ | 🔿  No_0_  🔿  No_0_ |  |
| 31. | Acted in a way that made you afraid that you might be physically hurt.  Please check all ages that apply.   \| 1 \| 2 \| 3 \| 4 \| 5 \| 6 \| 7 \| 8 \| 9 \| 10 \| 11 \| 12 \| 13 \| 14 \| 15 \| 16 \| 17 \| 18 \| \| --- \| --- \| --- \| --- \| --- \| --- \| --- \| --- \| --- \| --- \| --- \| --- \| --- \| --- \| --- \| --- \| --- \| --- \| \|  \|  \|  \|  \|  \|  \|  \|  \|  \|  \|  \|  \|  \|  \|  \|  \|  \|  \|   Please indicate ages when (if) the person doing this to you was a date.   \| 1 \| 2 \| 3 \| 4 \| 5 \| 6 \| 7 \| 8 \| 9 \| 10 \| 11 \| 12 \| 13 \| 14 \| 15 \| 16 \| 17 \| 18 \| \| --- \| --- \| --- \| --- \| --- \| --- \| --- \| --- \| --- \| --- \| --- \| --- \| --- \| --- \| --- \| --- \| --- \| --- \| \|  \|  \|  \|  \|  \|  \|  \|  \|  \|  \|  \|  \|  \|  \|  \|  \|  \|  \| | 🔿  Yes_1_  🔿  Yes_1_ | 🔿  No_0_  🔿  No_0_ |  |
| 32. | Threatened you in order to take your money or possessions.  Please check all ages that apply.   \| 1 \| 2 \| 3 \| 4 \| 5 \| 6 \| 7 \| 8 \| 9 \| 10 \| 11 \| 12 \| 13 \| 14 \| 15 \| 16 \| 17 \| 18 \| \| --- \| --- \| --- \| --- \| --- \| --- \| --- \| --- \| --- \| --- \| --- \| --- \| --- \| --- \| --- \| --- \| --- \| --- \| \|  \|  \|  \|  \|  \|  \|  \|  \|  \|  \|  \|  \|  \|  \|  \|  \|  \|  \|   Please indicate ages when (if) the person doing this to you was a date.   \| 1 \| 2 \| 3 \| 4 \| 5 \| 6 \| 7 \| 8 \| 9 \| 10 \| 11 \| 12 \| 13 \| 14 \| 15 \| 16 \| 17 \| 18 \| \| --- \| --- \| --- \| --- \| --- \| --- \| --- \| --- \| --- \| --- \| --- \| --- \| --- \| --- \| --- \| --- \| --- \| --- \| \|  \|  \|  \|  \|  \|  \|  \|  \|  \|  \|  \|  \|  \|  \|  \|  \|  \|  \| | 🔿  Yes_1_  🔿  Yes_1_ | 🔿  No_0_  🔿  No_0_ |  |
| 33. | Forced or threatened you to do things that you did not want to do.  Please check all ages that apply.   \| 1 \| 2 \| 3 \| 4 \| 5 \| 6 \| 7 \| 8 \| 9 \| 10 \| 11 \| 12 \| 13 \| 14 \| 15 \| 16 \| 17 \| 18 \| \| --- \| --- \| --- \| --- \| --- \| --- \| --- \| --- \| --- \| --- \| --- \| --- \| --- \| --- \| --- \| --- \| --- \| --- \| \|  \|  \|  \|  \|  \|  \|  \|  \|  \|  \|  \|  \|  \|  \|  \|  \|  \|  \|   If yes, please describe examples:   \|  \| \| --- \|   Please indicate ages when (if) the person doing this to you was a date.   \| 1 \| 2 \| 3 \| 4 \| 5 \| 6 \| 7 \| 8 \| 9 \| 10 \| 11 \| 12 \| 13 \| 14 \| 15 \| 16 \| 17 \| 18 \| \| --- \| --- \| --- \| --- \| --- \| --- \| --- \| --- \| --- \| --- \| --- \| --- \| --- \| --- \| --- \| --- \| --- \| --- \| \|  \|  \|  \|  \|  \|  \|  \|  \|  \|  \|  \|  \|  \|  \|  \|  \|  \|  \| | 🔿  Yes_1_  🔿  Yes_1_ | 🔿  No_0_  🔿  No_0_ |  |

| 34. | Intentionally pushed, grabbed, shoved, slapped, pinched, punched, or kicked you.  Please check all ages that apply.   \| 1 \| 2 \| 3 \| 4 \| 5 \| 6 \| 7 \| 8 \| 9 \| 10 \| 11 \| 12 \| 13 \| 14 \| 15 \| 16 \| 17 \| 18 \| \| --- \| --- \| --- \| --- \| --- \| --- \| --- \| --- \| --- \| --- \| --- \| --- \| --- \| --- \| --- \| --- \| --- \| --- \| \|  \|  \|  \|  \|  \|  \|  \|  \|  \|  \|  \|  \|  \|  \|  \|  \|  \|  \|   Please indicate ages when (if) the person doing this to you was a date.   \| 1 \| 2 \| 3 \| 4 \| 5 \| 6 \| 7 \| 8 \| 9 \| 10 \| 11 \| 12 \| 13 \| 14 \| 15 \| 16 \| 17 \| 18 \| \| --- \| --- \| --- \| --- \| --- \| --- \| --- \| --- \| --- \| --- \| --- \| --- \| --- \| --- \| --- \| --- \| --- \| --- \| \|  \|  \|  \|  \|  \|  \|  \|  \|  \|  \|  \|  \|  \|  \|  \|  \|  \|  \| | 🔿  Yes_1_  🔿  Yes_1_ | 🔿  No_0_  🔿  No_0_ |  |
| --- | --- | --- | --- | --- | --- | --- | --- | --- | --- | --- | --- | --- | --- | --- | --- | --- | --- | --- | --- | --- | --- | --- | --- | --- | --- | --- | --- | --- | --- | --- | --- | --- | --- | --- | --- | --- | --- | --- | --- | --- | --- | --- | --- | --- | --- | --- | --- | --- | --- | --- | --- | --- | --- | --- | --- | --- | --- | --- | --- | --- | --- | --- | --- | --- | --- | --- | --- | --- | --- | --- | --- | --- | --- | --- | --- | --- |
| 35. | Hit you so hard, or intentionally harmed you in some way, that you received or should have received medical attention.  Please check all ages that apply.   \| 1 \| 2 \| 3 \| 4 \| 5 \| 6 \| 7 \| 8 \| 9 \| 10 \| 11 \| 12 \| 13 \| 14 \| 15 \| 16 \| 17 \| 18 \| \| --- \| --- \| --- \| --- \| --- \| --- \| --- \| --- \| --- \| --- \| --- \| --- \| --- \| --- \| --- \| --- \| --- \| --- \| \|  \|  \|  \|  \|  \|  \|  \|  \|  \|  \|  \|  \|  \|  \|  \|  \|  \|  \|   Please indicate ages when (if) the person doing this to you was a date.   \| 1 \| 2 \| 3 \| 4 \| 5 \| 6 \| 7 \| 8 \| 9 \| 10 \| 11 \| 12 \| 13 \| 14 \| 15 \| 16 \| 17 \| 18 \| \| --- \| --- \| --- \| --- \| --- \| --- \| --- \| --- \| --- \| --- \| --- \| --- \| --- \| --- \| --- \| --- \| --- \| --- \| \|  \|  \|  \|  \|  \|  \|  \|  \|  \|  \|  \|  \|  \|  \|  \|  \|  \|  \| | 🔿  Yes_1_  🔿  Yes_1_ | 🔿  No_0_  🔿  No_0_ |  |
| 36. | Forced you to engage in sexual activity against your will.  Please check all ages that apply.   \| 1 \| 2 \| 3 \| 4 \| 5 \| 6 \| 7 \| 8 \| 9 \| 10 \| 11 \| 12 \| 13 \| 14 \| 15 \| 16 \| 17 \| 18 \| \| --- \| --- \| --- \| --- \| --- \| --- \| --- \| --- \| --- \| --- \| --- \| --- \| --- \| --- \| --- \| --- \| --- \| --- \| \|  \|  \|  \|  \|  \|  \|  \|  \|  \|  \|  \|  \|  \|  \|  \|  \|  \|  \|   Please indicate ages when (if) the person doing this to you was a date.   \| 1 \| 2 \| 3 \| 4 \| 5 \| 6 \| 7 \| 8 \| 9 \| 10 \| 11 \| 12 \| 13 \| 14 \| 15 \| 16 \| 17 \| 18 \| \| --- \| --- \| --- \| --- \| --- \| --- \| --- \| --- \| --- \| --- \| --- \| --- \| --- \| --- \| --- \| --- \| --- \| --- \| \|  \|  \|  \|  \|  \|  \|  \|  \|  \|  \|  \|  \|  \|  \|  \|  \|  \|  \| | 🔿  Yes_1_  🔿  Yes_1_ | 🔿  No_0_  🔿  No_0_ |  |
| 37. | Forced you to do things sexually that you did not want to do.  Please check all ages that apply.   \| 1 \| 2 \| 3 \| 4 \| 5 \| 6 \| 7 \| 8 \| 9 \| 10 \| 11 \| 12 \| 13 \| 14 \| 15 \| 16 \| 17 \| 18 \| \| --- \| --- \| --- \| --- \| --- \| --- \| --- \| --- \| --- \| --- \| --- \| --- \| --- \| --- \| --- \| --- \| --- \| --- \| \|  \|  \|  \|  \|  \|  \|  \|  \|  \|  \|  \|  \|  \|  \|  \|  \|  \|  \|   Please indicate ages when (if) the person doing this to you was a date.   \| 1 \| 2 \| 3 \| 4 \| 5 \| 6 \| 7 \| 8 \| 9 \| 10 \| 11 \| 12 \| 13 \| 14 \| 15 \| 16 \| 17 \| 18 \| \| --- \| --- \| --- \| --- \| --- \| --- \| --- \| --- \| --- \| --- \| --- \| --- \| --- \| --- \| --- \| --- \| --- \| --- \| \|  \|  \|  \|  \|  \|  \|  \|  \|  \|  \|  \|  \|  \|  \|  \|  \|  \|  \| | 🔿  Yes_1_  🔿  Yes_1_ | 🔿  No_0_  🔿  No_0_ |  |

|  | Please indicate if the following happened during your childhood (first 18 years of your life). Please provide your best estimates of your age at the time(s) of occurrence.  **Please check all ages that apply.** | | |  | |  | |
| --- | --- | --- | --- | --- | --- | --- | --- |
| 38. | | You felt that your mother or other important maternal figure was present in the household but emotionally unavailable to you for a variety of reasons like drugs, alcohol, workaholic, having an affair, heedlessly pursuing their own goals.  Please check all ages that apply.   \| 1 \| 2 \| 3 \| 4 \| 5 \| 6 \| 7 \| 8 \| 9 \| 10 \| 11 \| 12 \| 13 \| 14 \| 15 \| 16 \| 17 \| 18 \| \| --- \| --- \| --- \| --- \| --- \| --- \| --- \| --- \| --- \| --- \| --- \| --- \| --- \| --- \| --- \| --- \| --- \| --- \| \|  \|  \|  \|  \|  \|  \|  \|  \|  \|  \|  \|  \|  \|  \|  \|  \|  \|  \| | 🔿  Yes_1_ | | 🔿  No_0_ | |  |
| 39. | | You felt that your father or other important paternal figure was present in the household but emotionally unavailable to you for a variety of reasons like drugs, alcohol, workaholic, having an affair, heedlessly pursuing their own goals.  Please check all ages that apply.   \| 1 \| 2 \| 3 \| 4 \| 5 \| 6 \| 7 \| 8 \| 9 \| 10 \| 11 \| 12 \| 13 \| 14 \| 15 \| 16 \| 17 \| 18 \| \| --- \| --- \| --- \| --- \| --- \| --- \| --- \| --- \| --- \| --- \| --- \| --- \| --- \| --- \| --- \| --- \| --- \| --- \| \|  \|  \|  \|  \|  \|  \|  \|  \|  \|  \|  \|  \|  \|  \|  \|  \|  \|  \| | 🔿  Yes_1_ | | 🔿  No_0_ | |  |
| 40. | | You felt that your mother or other important maternal figure was emotionally unavailable to you for a variety of reasons like military service, taking care of a sick relative, in school, business necessity.  Please check all ages that apply.   \| 1 \| 2 \| 3 \| 4 \| 5 \| 6 \| 7 \| 8 \| 9 \| 10 \| 11 \| 12 \| 13 \| 14 \| 15 \| 16 \| 17 \| 18 \| \| --- \| --- \| --- \| --- \| --- \| --- \| --- \| --- \| --- \| --- \| --- \| --- \| --- \| --- \| --- \| --- \| --- \| --- \| \|  \|  \|  \|  \|  \|  \|  \|  \|  \|  \|  \|  \|  \|  \|  \|  \|  \|  \| | 🔿  Yes_1_ | | 🔿  No_0_ | |  |
| 41. | | You felt that your father or other important paternal figure was emotionally unavailable to you for a variety of reasons like military service, taking care of a sick relative, in school, business necessity.  Please check all ages that apply.   \| 1 \| 2 \| 3 \| 4 \| 5 \| 6 \| 7 \| 8 \| 9 \| 10 \| 11 \| 12 \| 13 \| 14 \| 15 \| 16 \| 17 \| 18 \| \| --- \| --- \| --- \| --- \| --- \| --- \| --- \| --- \| --- \| --- \| --- \| --- \| --- \| --- \| --- \| --- \| --- \| --- \| \|  \|  \|  \|  \|  \|  \|  \|  \|  \|  \|  \|  \|  \|  \|  \|  \|  \|  \| | 🔿  Yes_1_ | | 🔿  No_0_ | |  |
| 42. | | A parent or other important parental figure was very difficult to please.  Please check all ages that apply.   \| 1 \| 2 \| 3 \| 4 \| 5 \| 6 \| 7 \| 8 \| 9 \| 10 \| 11 \| 12 \| 13 \| 14 \| 15 \| 16 \| 17 \| 18 \| \| --- \| --- \| --- \| --- \| --- \| --- \| --- \| --- \| --- \| --- \| --- \| --- \| --- \| --- \| --- \| --- \| --- \| --- \| \|  \|  \|  \|  \|  \|  \|  \|  \|  \|  \|  \|  \|  \|  \|  \|  \|  \|  \| | 🔿  Yes_1_ | | 🔿  No_0_ | |  |
| 43. | | A parent or other important parental figure did not have the time or interest to talk to you.  Please check all ages that apply.   \| 1 \| 2 \| 3 \| 4 \| 5 \| 6 \| 7 \| 8 \| 9 \| 10 \| 11 \| 12 \| 13 \| 14 \| 15 \| 16 \| 17 \| 18 \| \| --- \| --- \| --- \| --- \| --- \| --- \| --- \| --- \| --- \| --- \| --- \| --- \| --- \| --- \| --- \| --- \| --- \| --- \| \|  \|  \|  \|  \|  \|  \|  \|  \|  \|  \|  \|  \|  \|  \|  \|  \|  \|  \| | 🔿  Yes_1_ | | 🔿  No_0_ | |  |
| 44. | | One or more individuals in your family made you feel loved.  Please check all ages that apply.   \| 1 \| 2 \| 3 \| 4 \| 5 \| 6 \| 7 \| 8 \| 9 \| 10 \| 11 \| 12 \| 13 \| 14 \| 15 \| 16 \| 17 \| 18 \| \| --- \| --- \| --- \| --- \| --- \| --- \| --- \| --- \| --- \| --- \| --- \| --- \| --- \| --- \| --- \| --- \| --- \| --- \| \|  \|  \|  \|  \|  \|  \|  \|  \|  \|  \|  \|  \|  \|  \|  \|  \|  \|  \|   Who? (e.g. mother, aunt, maternal grandfather)   \|  \| \| --- \| | 🔿  Yes_1_ | | 🔿  No_0_ | |  |

| 45. | One or more individuals in your family helped you feel important or special.  Please check all ages that apply.   \| 1 \| 2 \| 3 \| 4 \| 5 \| 6 \| 7 \| 8 \| 9 \| 10 \| 11 \| 12 \| 13 \| 14 \| 15 \| 16 \| 17 \| 18 \| \| --- \| --- \| --- \| --- \| --- \| --- \| --- \| --- \| --- \| --- \| --- \| --- \| --- \| --- \| --- \| --- \| --- \| --- \| \|  \|  \|  \|  \|  \|  \|  \|  \|  \|  \|  \|  \|  \|  \|  \|  \|  \|  \|   Who? (e.g. mother, aunt, maternal grandfather)   \|  \| \| --- \| | 🔿  Yes_1_ | 🔿  No_0_ |  |
| --- | --- | --- | --- | --- | --- | --- | --- | --- | --- | --- | --- | --- | --- | --- | --- | --- | --- | --- | --- | --- | --- | --- | --- | --- | --- | --- | --- | --- | --- | --- | --- | --- | --- | --- | --- | --- | --- | --- | --- | --- | --- |
| 46. | One or more individuals in your family were there to take care of you and protect you.  Please check all ages that apply.   \| 1 \| 2 \| 3 \| 4 \| 5 \| 6 \| 7 \| 8 \| 9 \| 10 \| 11 \| 12 \| 13 \| 14 \| 15 \| 16 \| 17 \| 18 \| \| --- \| --- \| --- \| --- \| --- \| --- \| --- \| --- \| --- \| --- \| --- \| --- \| --- \| --- \| --- \| --- \| --- \| --- \| \|  \|  \|  \|  \|  \|  \|  \|  \|  \|  \|  \|  \|  \|  \|  \|  \|  \|  \|   Who? (e.g. mother, aunt, maternal grandfather)   \|  \| \| --- \| | 🔿  Yes_1_ | 🔿  No_0_ |  |
| 47. | One or more individuals in your family were there to take you to the doctor or Emergency Room if the need ever arose.  Please check all ages that apply.   \| 1 \| 2 \| 3 \| 4 \| 5 \| 6 \| 7 \| 8 \| 9 \| 10 \| 11 \| 12 \| 13 \| 14 \| 15 \| 16 \| 17 \| 18 \| \| --- \| --- \| --- \| --- \| --- \| --- \| --- \| --- \| --- \| --- \| --- \| --- \| --- \| --- \| --- \| --- \| --- \| --- \| \|  \|  \|  \|  \|  \|  \|  \|  \|  \|  \|  \|  \|  \|  \|  \|  \|  \|  \|   Who? (e.g. mother, aunt, maternal grandfather)   \|  \| \| --- \| | 🔿  Yes_1_ | 🔿  No_0_ |  |
| 48. | One or more individuals in your family would help you with your homework, or to get ready for school.  Please check all ages that apply.   \| 1 \| 2 \| 3 \| 4 \| 5 \| 6 \| 7 \| 8 \| 9 \| 10 \| 11 \| 12 \| 13 \| 14 \| 15 \| 16 \| 17 \| 18 \| \| --- \| --- \| --- \| --- \| --- \| --- \| --- \| --- \| --- \| --- \| --- \| --- \| --- \| --- \| --- \| --- \| --- \| --- \| \|  \|  \|  \|  \|  \|  \|  \|  \|  \|  \|  \|  \|  \|  \|  \|  \|  \|  \| | 🔿  Yes_1_ | 🔿  No_0_ |  |

|  | **Please indicate if the following statements were true about you and your family during your childhood, and your age at the time(s) you felt this to be true.**  **Please check all ages that apply.** | | |  | |  | |
| --- | --- | --- | --- | --- | --- | --- | --- |
| 49. | | You didn’t have enough to eat.  Please check all ages that apply.   \| 1 \| 2 \| 3 \| 4 \| 5 \| 6 \| 7 \| 8 \| 9 \| 10 \| 11 \| 12 \| 13 \| 14 \| 15 \| 16 \| 17 \| 18 \| \| --- \| --- \| --- \| --- \| --- \| --- \| --- \| --- \| --- \| --- \| --- \| --- \| --- \| --- \| --- \| --- \| --- \| --- \| \|  \|  \|  \|  \|  \|  \|  \|  \|  \|  \|  \|  \|  \|  \|  \|  \|  \|  \| | 🔿  Yes_1_ | | 🔿  No_0_ | |  |
| 50. | | You had to wear dirty clothes.  Please check all ages that apply.   \| 1 \| 2 \| 3 \| 4 \| 5 \| 6 \| 7 \| 8 \| 9 \| 10 \| 11 \| 12 \| 13 \| 14 \| 15 \| 16 \| 17 \| 18 \| \| --- \| --- \| --- \| --- \| --- \| --- \| --- \| --- \| --- \| --- \| --- \| --- \| --- \| --- \| --- \| --- \| --- \| --- \| \|  \|  \|  \|  \|  \|  \|  \|  \|  \|  \|  \|  \|  \|  \|  \|  \|  \|  \| | 🔿  Yes_1_ | | 🔿  No_0_ | |  |
| 51. | | You were left unsupervised at an age or in situations when you should have been supervised.  Please check all ages that apply.   \| 1 \| 2 \| 3 \| 4 \| 5 \| 6 \| 7 \| 8 \| 9 \| 10 \| 11 \| 12 \| 13 \| 14 \| 15 \| 16 \| 17 \| 18 \| \| --- \| --- \| --- \| --- \| --- \| --- \| --- \| --- \| --- \| --- \| --- \| --- \| --- \| --- \| --- \| --- \| --- \| --- \| \|  \|  \|  \|  \|  \|  \|  \|  \|  \|  \|  \|  \|  \|  \|  \|  \|  \|  \| | 🔿  Yes_1_ | | 🔿  No_0_ | |  |
| 52. | | You felt that you had to shoulder adult responsibilities.  Please check all ages that apply.   \| 1 \| 2 \| 3 \| 4 \| 5 \| 6 \| 7 \| 8 \| 9 \| 10 \| 11 \| 12 \| 13 \| 14 \| 15 \| 16 \| 17 \| 18 \| \| --- \| --- \| --- \| --- \| --- \| --- \| --- \| --- \| --- \| --- \| --- \| --- \| --- \| --- \| --- \| --- \| --- \| --- \| \|  \|  \|  \|  \|  \|  \|  \|  \|  \|  \|  \|  \|  \|  \|  \|  \|  \|  \| | 🔿  Yes_1_ | | 🔿  No_0_ | |  |
| 53. | | You felt that your family was under severe financial pressure.  Please check all ages that apply.   \| 1 \| 2 \| 3 \| 4 \| 5 \| 6 \| 7 \| 8 \| 9 \| 10 \| 11 \| 12 \| 13 \| 14 \| 15 \| 16 \| 17 \| 18 \| \| --- \| --- \| --- \| --- \| --- \| --- \| --- \| --- \| --- \| --- \| --- \| --- \| --- \| --- \| --- \| --- \| --- \| --- \| \|  \|  \|  \|  \|  \|  \|  \|  \|  \|  \|  \|  \|  \|  \|  \|  \|  \|  \| | 🔿  Yes_1_ | | 🔿  No_0_ | |  |
| 54. | | One or more individuals kept important secrets or facts from you.  Please check all ages that apply.   \| 1 \| 2 \| 3 \| 4 \| 5 \| 6 \| 7 \| 8 \| 9 \| 10 \| 11 \| 12 \| 13 \| 14 \| 15 \| 16 \| 17 \| 18 \| \| --- \| --- \| --- \| --- \| --- \| --- \| --- \| --- \| --- \| --- \| --- \| --- \| --- \| --- \| --- \| --- \| --- \| --- \| \|  \|  \|  \|  \|  \|  \|  \|  \|  \|  \|  \|  \|  \|  \|  \|  \|  \|  \| | 🔿  Yes_1_ | | 🔿  No_0_ | |  |
| 55. | | Your parents were separated.  Please check all ages that apply.   \| 1 \| 2 \| 3 \| 4 \| 5 \| 6 \| 7 \| 8 \| 9 \| 10 \| 11 \| 12 \| 13 \| 14 \| 15 \| 16 \| 17 \| 18 \| \| --- \| --- \| --- \| --- \| --- \| --- \| --- \| --- \| --- \| --- \| --- \| --- \| --- \| --- \| --- \| --- \| --- \| --- \| \|  \|  \|  \|  \|  \|  \|  \|  \|  \|  \|  \|  \|  \|  \|  \|  \|  \|  \| | 🔿  Yes_1_ | | 🔿  No_0_ | |  |
| 56. | | People in your family looked out for each other.  Please check all ages that apply.   \| 1 \| 2 \| 3 \| 4 \| 5 \| 6 \| 7 \| 8 \| 9 \| 10 \| 11 \| 12 \| 13 \| 14 \| 15 \| 16 \| 17 \| 18 \| \| --- \| --- \| --- \| --- \| --- \| --- \| --- \| --- \| --- \| --- \| --- \| --- \| --- \| --- \| --- \| --- \| --- \| --- \| \|  \|  \|  \|  \|  \|  \|  \|  \|  \|  \|  \|  \|  \|  \|  \|  \|  \|  \| | 🔿  Yes_1_ | | 🔿  No_0_ | |  |
| 57. | | People in your family felt close to each other.  Please check all ages that apply.   \| 1 \| 2 \| 3 \| 4 \| 5 \| 6 \| 7 \| 8 \| 9 \| 10 \| 11 \| 12 \| 13 \| 14 \| 15 \| 16 \| 17 \| 18 \| \| --- \| --- \| --- \| --- \| --- \| --- \| --- \| --- \| --- \| --- \| --- \| --- \| --- \| --- \| --- \| --- \| --- \| --- \| \|  \|  \|  \|  \|  \|  \|  \|  \|  \|  \|  \|  \|  \|  \|  \|  \|  \|  \| | 🔿  Yes_1_ | | 🔿  No_0_ | |  |
| 58. | | Your family was a source of strength and support.  Please check all ages that apply.   \| 1 \| 2 \| 3 \| 4 \| 5 \| 6 \| 7 \| 8 \| 9 \| 10 \| 11 \| 12 \| 13 \| 14 \| 15 \| 16 \| 17 \| 18 \| \| --- \| --- \| --- \| --- \| --- \| --- \| --- \| --- \| --- \| --- \| --- \| --- \| --- \| --- \| --- \| --- \| --- \| --- \| \|  \|  \|  \|  \|  \|  \|  \|  \|  \|  \|  \|  \|  \|  \|  \|  \|  \|  \| | 🔿  Yes_1_ | | 🔿  No_0_ | |  |
