# Supplementary material for: Polish adaptation of ‘Maltreatment and Abuse Chronology of Exposure’ scale
Source: PLoS One. 2025 Apr 9;20(4):e0321046. doi: 10.1371/journal.pone.0321046 (PMC11981210; doi:10.1371/journal.pone.0321046)
Supplement: S2 File — (DOCX) [file pone.0321046.s002.docx]

**MACE-58(PL)**

**(Maltreatment and Abuse Chronology of Exposure – wersja polska)**

Autorzy adaptacji: M. Chęć, S. Michałowska, K. Rachubińska, K. Konieczny, A. Samochowiec

Autorzy wersji oryginalnej: M. H. Teicher, A. Parigger

Płeć: ______________________ Data urodzenia: _________________________________

Zawód:__________________________________________ Wykształcenie: __________________________________

| **Czasami rodzice, przybrani rodzice czy inni dorośli mieszkający w jednym domu robią coś,**  **co może krzywdzić innych.**  **Jeśli któraś z poniższych sytuacji zdarzyła się w Twoim domu, w pierwszych 18 latach Twojego życia,**  **zaznacz TAK.**  **Postaraj się także jak najlepiej określić i zaznaczyć wszystkie lata Twojego życia, w których sytuacje te miały miejsce.** | | |
| --- | --- | --- |
| *Przykład 1.* Używano wobec Ciebie wulgarnego języka, wyzwisk, wypowiadano obraźliwe  treści, mówiąc, że jesteś “gruby/-a”, “brzydki/-a”, “głupi/-a” częściej niż kilka razy w roku.  *Jeśli Twój ojciec używał wobec Ciebie wulgaryzmów kiedy miałeś/-aś 6-8 lat, matka*  *obrażała Cię kiedy miałeś/-aś 8-10 lat, zaś kiedy miałeś/-aś 17 lat mieszkający z Wami*  *konkubent matki wyzywał Cię, powinieneś/powinnaś zaznaczyć następujące kratki:*   \| 1 \| 2 \| 3 \| 4 \| 5 \| 6 \| 7 \| 8 \| 9 \| 10 \| 11 \| 12 \| 13 \| 14 \| 15 \| 16 \| 17 \| 18 \| \| --- \| --- \| --- \| --- \| --- \| --- \| --- \| --- \| --- \| --- \| --- \| --- \| --- \| --- \| --- \| --- \| --- \| --- \| \|  \|  \|  \|  \|  \| 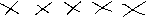 \|  \|  \|  \|  \|  \|  \|  \|  \|  \|  \| 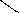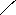 \|  \| | ⚫  Tak | 🔿  Nie |
| 1. Używano wobec Ciebie wulgarnego języka, wyzwisk, wypowiadano obraźliwe treści, mówiąc,   że jesteś “gruby/-a”, “brzydki/-a”, “głupi/-a” częściej niż kilka razy w roku.  Zaznacz, ile miałeś/miałaś lat w każdym przypadku, gdy dochodziło do takiego zdarzenia.   \| 1 \| 2 \| 3 \| 4 \| 5 \| 6 \| 7 \| 8 \| 9 \| 10 \| 11 \| 12 \| 13 \| 14 \| 15 \| 16 \| 17 \| 18 \| \| --- \| --- \| --- \| --- \| --- \| --- \| --- \| --- \| --- \| --- \| --- \| --- \| --- \| --- \| --- \| --- \| --- \| --- \| \|  \|  \|  \|  \|  \|  \|  \|  \|  \|  \|  \|  \|  \|  \|  \|  \|  \|  \| | 🔿  Tak_1_ | 🔿  Nie_0_ |
| 1. Wypowiadano bolesne treści, które powodowały, że było Ci przykro, lub czułeś/-aś się   zawstydzony/-a czy upokorzony/-a częściej niż kilka razy w roku.  Zaznacz, ile miałeś/miałaś lat w każdym przypadku, gdy dochodziło do takiego zdarzenia.   \| 1 \| 2 \| 3 \| 4 \| 5 \| 6 \| 7 \| 8 \| 9 \| 10 \| 11 \| 12 \| 13 \| 14 \| 15 \| 16 \| 17 \| 18 \| \| --- \| --- \| --- \| --- \| --- \| --- \| --- \| --- \| --- \| --- \| --- \| --- \| --- \| --- \| --- \| --- \| --- \| --- \| \|  \|  \|  \|  \|  \|  \|  \|  \|  \|  \|  \|  \|  \|  \|  \|  \|  \|  \| | 🔿  Tak_1_ | 🔿  Nie_0_ |
| 1. Krzyczano na Ciebie częściej niż kilka razy w roku.   Zaznacz, ile miałeś/miałaś lat w każdym przypadku, gdy dochodziło do takiego zdarzenia.   \| 1 \| 2 \| 3 \| 4 \| 5 \| 6 \| 7 \| 8 \| 9 \| 10 \| 11 \| 12 \| 13 \| 14 \| 15 \| 16 \| 17 \| 18 \| \| --- \| --- \| --- \| --- \| --- \| --- \| --- \| --- \| --- \| --- \| --- \| --- \| --- \| --- \| --- \| --- \| --- \| --- \| \|  \|  \|  \|  \|  \|  \|  \|  \|  \|  \|  \|  \|  \|  \|  \|  \|  \|  \| | 🔿  Tak_1_ | 🔿  Nie_0_ |
| 1. Zachowywano się w sposób, który sprawiał, że obawiałeś/-aś się, że możesz doznać krzywdy fizycznej.   Zaznacz, ile miałeś/miałaś lat w każdym przypadku, gdy dochodziło do takiego zdarzenia.   \| 1 \| 2 \| 3 \| 4 \| 5 \| 6 \| 7 \| 8 \| 9 \| 10 \| 11 \| 12 \| 13 \| 14 \| 15 \| 16 \| 17 \| 18 \| \| --- \| --- \| --- \| --- \| --- \| --- \| --- \| --- \| --- \| --- \| --- \| --- \| --- \| --- \| --- \| --- \| --- \| --- \| \|  \|  \|  \|  \|  \|  \|  \|  \|  \|  \|  \|  \|  \|  \|  \|  \|  \|  \| | 🔿  Tak_1_ | 🔿  Nie_0_ |
| 1. Grożono zostawieniem lub porzuceniem Ciebie.   Zaznacz, ile miałeś/miałaś lat w każdym przypadku, gdy dochodziło do takiego zdarzenia.   \| 1 \| 2 \| 3 \| 4 \| 5 \| 6 \| 7 \| 8 \| 9 \| 10 \| 11 \| 12 \| 13 \| 14 \| 15 \| 16 \| 17 \| 18 \| \| --- \| --- \| --- \| --- \| --- \| --- \| --- \| --- \| --- \| --- \| --- \| --- \| --- \| --- \| --- \| --- \| --- \| --- \| \|  \|  \|  \|  \|  \|  \|  \|  \|  \|  \|  \|  \|  \|  \|  \|  \|  \|  \|     Wskaż, czy odczuwałeś/-aś wówczas bezradność i/lub przerażenie. | 🔿  Tak_1_  ❒ Bezradność | 🔿  Nie_0_  ❒ Przerażenie |
| 1. Zamykano Cię w szafie, na strychu, w piwnicy lub garażu.   Zaznacz, ile miałeś/miałaś lat w każdym przypadku, gdy dochodziło do takiego zdarzenia.   \| 1 \| 2 \| 3 \| 4 \| 5 \| 6 \| 7 \| 8 \| 9 \| 10 \| 11 \| 12 \| 13 \| 14 \| 15 \| 16 \| 17 \| 18 \| \| --- \| --- \| --- \| --- \| --- \| --- \| --- \| --- \| --- \| --- \| --- \| --- \| --- \| --- \| --- \| --- \| --- \| --- \| \|  \|  \|  \|  \|  \|  \|  \|  \|  \|  \|  \|  \|  \|  \|  \|  \|  \|  \|     Wskaż, czy odczuwałeś/-aś wówczas bezradność i/lub przerażenie. | 🔿  Tak_1_  ❒ Bezradność | 🔿  Nie_0_  ❒ Przerażenie |
| 1. Celowo popychano Cię, szarpano, szczypano, uderzano otwartą dłonią lub pięścią bądź kopano.   Zaznacz, ile miałeś/miałaś lat w każdym przypadku, gdy dochodziło do takiego zdarzenia.   \| 1 \| 2 \| 3 \| 4 \| 5 \| 6 \| 7 \| 8 \| 9 \| 10 \| 11 \| 12 \| 13 \| 14 \| 15 \| 16 \| 17 \| 18 \| \| --- \| --- \| --- \| --- \| --- \| --- \| --- \| --- \| --- \| --- \| --- \| --- \| --- \| --- \| --- \| --- \| --- \| --- \| \|  \|  \|  \|  \|  \|  \|  \|  \|  \|  \|  \|  \|  \|  \|  \|  \|  \|  \|     Wskaż, czy odczuwałeś/-aś wówczas bezradność i/lub przerażenie. | 🔿  Tak_1_  ❒ Bezradność | 🔿  Nie_0_  ❒ Przerażenie |
| 1. Bito Cię tak mocno, że pozostawiało to na Twoim ciele ślady na dłużej niż kilka minut.   Zaznacz, ile miałeś/miałaś lat w każdym przypadku, gdy dochodziło do takiego zdarzenia.   \| 1 \| 2 \| 3 \| 4 \| 5 \| 6 \| 7 \| 8 \| 9 \| 10 \| 11 \| 12 \| 13 \| 14 \| 15 \| 16 \| 17 \| 18 \| \| --- \| --- \| --- \| --- \| --- \| --- \| --- \| --- \| --- \| --- \| --- \| --- \| --- \| --- \| --- \| --- \| --- \| --- \| \|  \|  \|  \|  \|  \|  \|  \|  \|  \|  \|  \|  \|  \|  \|  \|  \|  \|  \|     Wskaż, czy odczuwałeś/-aś wówczas bezradność i/lub przerażenie. | 🔿  Tak_1_  ❒ Bezradność | 🔿  Nie_0_  ❒ Przerażenie |
| 1. Dawano Ci klapsy w pośladki, ręce lub nogi.   Zaznacz, ile miałeś/miałaś lat w każdym przypadku, gdy dochodziło do takiego zdarzenia.   \| 1 \| 2 \| 3 \| 4 \| 5 \| 6 \| 7 \| 8 \| 9 \| 10 \| 11 \| 12 \| 13 \| 14 \| 15 \| 16 \| 17 \| 18 \| \| --- \| --- \| --- \| --- \| --- \| --- \| --- \| --- \| --- \| --- \| --- \| --- \| --- \| --- \| --- \| --- \| --- \| --- \| \|  \|  \|  \|  \|  \|  \|  \|  \|  \|  \|  \|  \|  \|  \|  \|  \|  \|  \|   Wskaż, czy odczuwałeś/-aś wówczas bezradność i/lub przerażenie. | 🔿  Tak_1_  ❒ Bezradność | 🔿  Nie_0_  ❒ Przerażenie |
| 1. Dawano Ci klapsy w nagie pośladki.   Zaznacz, ile miałeś/miałaś lat w każdym przypadku, gdy dochodziło do takiego zdarzenia.   \| 1 \| 2 \| 3 \| 4 \| 5 \| 6 \| 7 \| 8 \| 9 \| 10 \| 11 \| 12 \| 13 \| 14 \| 15 \| 16 \| 17 \| 18 \| \| --- \| --- \| --- \| --- \| --- \| --- \| --- \| --- \| --- \| --- \| --- \| --- \| --- \| --- \| --- \| --- \| --- \| --- \| \|  \|  \|  \|  \|  \|  \|  \|  \|  \|  \|  \|  \|  \|  \|  \|  \|  \|  \|   Wskaż, czy odczuwałeś/-aś wówczas bezradność i/lub przerażenie. | 🔿  Tak_1_  ❒ Bezradność | 🔿  Nie_0_  ❒ Przerażenie |
| 1. Bito Cię przy użyciu jakiegoś przedmiotu, np. paska, szczotki, łopatki, kija, pręta itp.   Zaznacz, ile miałeś/miałaś lat w każdym przypadku, gdy dochodziło do takiego zdarzenia.   \| 1 \| 2 \| 3 \| 4 \| 5 \| 6 \| 7 \| 8 \| 9 \| 10 \| 11 \| 12 \| 13 \| 14 \| 15 \| 16 \| 17 \| 18 \| \| --- \| --- \| --- \| --- \| --- \| --- \| --- \| --- \| --- \| --- \| --- \| --- \| --- \| --- \| --- \| --- \| --- \| --- \| \|  \|  \|  \|  \|  \|  \|  \|  \|  \|  \|  \|  \|  \|  \|  \|  \|  \|  \|   Wskaż, czy odczuwałeś/-aś wówczas bezradność i/lub przerażenie. | 🔿  Tak_1_  ❒ Bezradność | 🔿  Nie_0_  ❒ Przerażenie |
| 1. Dotykano Twoje ciało z podtekstem seksualnym.   Zaznacz, ile miałeś/miałaś lat w każdym przypadku, gdy dochodziło do takiego zdarzenia.   \| 1 \| 2 \| 3 \| 4 \| 5 \| 6 \| 7 \| 8 \| 9 \| 10 \| 11 \| 12 \| 13 \| 14 \| 15 \| 16 \| 17 \| 18 \| \| --- \| --- \| --- \| --- \| --- \| --- \| --- \| --- \| --- \| --- \| --- \| --- \| --- \| --- \| --- \| --- \| --- \| --- \| \|  \|  \|  \|  \|  \|  \|  \|  \|  \|  \|  \|  \|  \|  \|  \|  \|  \|  \|     Wskaż, czy odczuwałeś/-aś wówczas bezradność i/lub przerażenie. | 🔿  Tak_1_  ❒ Bezradność | 🔿  Nie_0_  ❒ Przerażenie |
| 1. Osoba dorosła kazała Ci dotykać jej ciało w sposób seksualny.   Zaznacz, ile miałeś/miałaś lat w każdym przypadku, gdy dochodziło do takiego zdarzenia.   \| 1 \| 2 \| 3 \| 4 \| 5 \| 6 \| 7 \| 8 \| 9 \| 10 \| 11 \| 12 \| 13 \| 14 \| 15 \| 16 \| 17 \| 18 \| \| --- \| --- \| --- \| --- \| --- \| --- \| --- \| --- \| --- \| --- \| --- \| --- \| --- \| --- \| --- \| --- \| --- \| --- \| \|  \|  \|  \|  \|  \|  \|  \|  \|  \|  \|  \|  \|  \|  \|  \|  \|  \|  \|   Wskaż, czy odczuwałeś/-aś wówczas bezradność i/lub przerażenie. | 🔿  Tak_1_  ❒ Bezradność | 🔿  Nie_0_  ❒ Przerażenie |

| **Czasami rodzice, przybrani rodzice lub inni dorośli mieszkający w Twoim domu wyrządzają krzywdę**  **Twojemu rodzeństwu (bratu, siostrze, przyrodniemu rodzeństwu).**  **Jeśli któraś z poniższych sytuacji zdarzyła się Twojemu bratu, siostrze, czy większej liczbie rodzeństwa**  **(także przyrodniego) w Twoim domu, w pierwszych osiemnastu latach Twojego życia, zaznacz TAK.**  **Postaraj się także jak najlepiej określić i zaznaczyć wszystkie lata Twojego życia, w których sytuacje te miały miejsce.** | | |
| --- | --- | --- |
| 1. Celowo popychano, szarpano, szczypano, uderzano otwartą dłonią lub pięścią bądź kopano Twoje rodzeństwo (rodzeństwo przyrodnie).   Zaznacz, ile miałeś/miałaś lat w każdym przypadku, gdy dochodziło do takiego zdarzenia.   \| 1 \| 2 \| 3 \| 4 \| 5 \| 6 \| 7 \| 8 \| 9 \| 10 \| 11 \| 12 \| 13 \| 14 \| 15 \| 16 \| 17 \| 18 \| \| --- \| --- \| --- \| --- \| --- \| --- \| --- \| --- \| --- \| --- \| --- \| --- \| --- \| --- \| --- \| --- \| --- \| --- \| \|  \|  \|  \|  \|  \|  \|  \|  \|  \|  \|  \|  \|  \|  \|  \|  \|  \|  \|   Wskaż, czy odczuwałeś/-aś wówczas bezradność i/lub przerażenie. | 🔿  Tak_1_  ❒ Bezradność | 🔿  Nie_0_  ❒ Przerażenie |
| 1. Bito Twoje rodzeństwo (rodzeństwo przyrodnie) tak mocno, że pozostawiało to na ich ciele ślady na dłużej niż kilka minut.   Zaznacz, ile miałeś/miałaś lat w każdym przypadku, gdy dochodziło do takiego zdarzenia.   \| 1 \| 2 \| 3 \| 4 \| 5 \| 6 \| 7 \| 8 \| 9 \| 10 \| 11 \| 12 \| 13 \| 14 \| 15 \| 16 \| 17 \| 18 \| \| --- \| --- \| --- \| --- \| --- \| --- \| --- \| --- \| --- \| --- \| --- \| --- \| --- \| --- \| --- \| --- \| --- \| --- \| \|  \|  \|  \|  \|  \|  \|  \|  \|  \|  \|  \|  \|  \|  \|  \|  \|  \|  \|   Wskaż, czy odczuwałeś/-aś wówczas bezradność i/lub przerażenie. | 🔿  Tak_1_  ❒ Bezradność | 🔿  Nie_0_  ❒ Przerażenie |
| 1. Bito Twoje rodzeństwo (rodzeństwo przyrodnie) lub w inny sposób krzywdzono tak mocno, że potrzebowali oni pomocy medycznej.   Zaznacz, ile miałeś/miałaś lat w każdym przypadku, gdy dochodziło do takiego zdarzenia.   \| 1 \| 2 \| 3 \| 4 \| 5 \| 6 \| 7 \| 8 \| 9 \| 10 \| 11 \| 12 \| 13 \| 14 \| 15 \| 16 \| 17 \| 18 \| \| --- \| --- \| --- \| --- \| --- \| --- \| --- \| --- \| --- \| --- \| --- \| --- \| --- \| --- \| --- \| --- \| --- \| --- \| \|  \|  \|  \|  \|  \|  \|  \|  \|  \|  \|  \|  \|  \|  \|  \|  \|  \|  \|   Wskaż, czy odczuwałeś/-aś wówczas bezradność i/lub przerażenie. | 🔿  Tak_1_  ❒ Bezradność | 🔿  Nie_0_  ❒ Przerażenie |
| 1. Kierowano do Twojego rodzeństwa (rodzeństwa przyrodniego) nieodpowiednie komentarze lub sugestie o charakterze seksualnym.   Zaznacz, ile miałeś/miałaś lat w każdym przypadku, gdy dochodziło do takiego zdarzenia.   \| 1 \| 2 \| 3 \| 4 \| 5 \| 6 \| 7 \| 8 \| 9 \| 10 \| 11 \| 12 \| 13 \| 14 \| 15 \| 16 \| 17 \| 18 \| \| --- \| --- \| --- \| --- \| --- \| --- \| --- \| --- \| --- \| --- \| --- \| --- \| --- \| --- \| --- \| --- \| --- \| --- \| \|  \|  \|  \|  \|  \|  \|  \|  \|  \|  \|  \|  \|  \|  \|  \|  \|  \|  \|   Wskaż, czy odczuwałeś/-aś wówczas bezradność i/lub przerażenie. | 🔿  Tak_1_  ❒ Bezradność | 🔿  Nie_0_  ❒ Przerażenie |
| 1. Grożono Twojemu rodzeństwu (rodzeństwu przyrodniemu) wyrządzeniem mu krzywdy.   Zaznacz, ile miałeś/miałaś lat w każdym przypadku, gdy dochodziło do takiego zdarzenia.   \| 1 \| 2 \| 3 \| 4 \| 5 \| 6 \| 7 \| 8 \| 9 \| 10 \| 11 \| 12 \| 13 \| 14 \| 15 \| 16 \| 17 \| 18 \| \| --- \| --- \| --- \| --- \| --- \| --- \| --- \| --- \| --- \| --- \| --- \| --- \| --- \| --- \| --- \| --- \| --- \| --- \| \|  \|  \|  \|  \|  \|  \|  \|  \|  \|  \|  \|  \|  \|  \|  \|  \|  \|  \|   Wskaż, czy odczuwałeś/-aś wówczas bezradność i/lub przerażenie. | 🔿  Tak_1_  ❒ Bezradność | 🔿  Nie_0_  ❒ Przerażenie |

| **Czasami dorośli lub osoby starsze, które NIE mieszkają w Twoim domu, mogą wyrządzić Ci krzywdę.**  **Jeśli któraś z poniższych sytuacji zdarzyła się w pierwszych osiemnastu latach Twojego życia,**  **zaznacz TAK.**  **Postaraj się także jak najlepiej określić i zaznaczyć wszystkie lata Twojego życia, w których sytuacje te miały miejsce.** | | |
| --- | --- | --- |
| 1. Kierowano do Ciebie nieodpowiednie komentarze lub sugestie o charakterze seksualnym.   Zaznacz, ile miałeś/miałaś lat w każdym przypadku, gdy dochodziło do takiego zdarzenia.   \| 1 \| 2 \| 3 \| 4 \| 5 \| 6 \| 7 \| 8 \| 9 \| 10 \| 11 \| 12 \| 13 \| 14 \| 15 \| 16 \| 17 \| 18 \| \| --- \| --- \| --- \| --- \| --- \| --- \| --- \| --- \| --- \| --- \| --- \| --- \| --- \| --- \| --- \| --- \| --- \| --- \| \|  \|  \|  \|  \|  \|  \|  \|  \|  \|  \|  \|  \|  \|  \|  \|  \|  \|  \|   Wskaż, czy odczuwałeś/-aś wówczas bezradność i/lub przerażenie. | 🔿  Tak_1_  ❒ Bezradność | 🔿  Nie_0_  ❒ Przerażenie |
| 1. Dotykano Twoje ciało w sposób seksualny.   Zaznacz, ile miałeś/miałaś lat w każdym przypadku, gdy dochodziło do takiego zdarzenia.   \| 1 \| 2 \| 3 \| 4 \| 5 \| 6 \| 7 \| 8 \| 9 \| 10 \| 11 \| 12 \| 13 \| 14 \| 15 \| 16 \| 17 \| 18 \| \| --- \| --- \| --- \| --- \| --- \| --- \| --- \| --- \| --- \| --- \| --- \| --- \| --- \| --- \| --- \| --- \| --- \| --- \| \|  \|  \|  \|  \|  \|  \|  \|  \|  \|  \|  \|  \|  \|  \|  \|  \|  \|  \|   Wskaż, czy odczuwałeś/-aś wówczas bezradność i/lub przerażenie. | 🔿  Tak_1_  ❒ Bezradność | 🔿  Nie_0_  ❒ Przerażenie |
| 1. Usiłowano odbyć z Tobą stosunek seksualny (oralny, analny lub waginalny).   Zaznacz, ile miałeś/miałaś lat w każdym przypadku, gdy dochodziło do takiego zdarzenia.   \| 1 \| 2 \| 3 \| 4 \| 5 \| 6 \| 7 \| 8 \| 9 \| 10 \| 11 \| 12 \| 13 \| 14 \| 15 \| 16 \| 17 \| 18 \| \| --- \| --- \| --- \| --- \| --- \| --- \| --- \| --- \| --- \| --- \| --- \| --- \| --- \| --- \| --- \| --- \| --- \| --- \| \|  \|  \|  \|  \|  \|  \|  \|  \|  \|  \|  \|  \|  \|  \|  \|  \|  \|  \|   Wskaż, czy odczuwałeś/-aś wówczas bezradność i/lub przerażenie. | 🔿  Tak_1_  ❒ Bezradność | 🔿  Nie_0_  ❒ Przerażenie |
| 1. Odbyto z Tobą stosunek seksualny (oralny, analny lub waginalny).   Zaznacz, ile miałeś/miałaś lat w każdym przypadku, gdy dochodziło do takiego zdarzenia.   \| 1 \| 2 \| 3 \| 4 \| 5 \| 6 \| 7 \| 8 \| 9 \| 10 \| 11 \| 12 \| 13 \| 14 \| 15 \| 16 \| 17 \| 18 \| \| --- \| --- \| --- \| --- \| --- \| --- \| --- \| --- \| --- \| --- \| --- \| --- \| --- \| --- \| --- \| --- \| --- \| --- \| \|  \|  \|  \|  \|  \|  \|  \|  \|  \|  \|  \|  \|  \|  \|  \|  \|  \|  \|   Wskaż, czy odczuwałeś/-aś wówczas bezradność i/lub przerażenie. | 🔿  Tak_1_  ❒ Bezradność | 🔿  Nie_0_  ❒ Przerażenie |

| **Czasami między dorosłymi mieszkającymi w Twoim domu (np. rodzicami, rodzicami zastępczymi,**  **partnerami czy dziadkami) wybuchają ostre kłótnie lub bójki.**  **Jeśli któraś z poniższych sytuacji zdarzyła się w pierwszych osiemnastu latach Twojego życia,**  **zaznacz TAK.**  **Postaraj się także jak najlepiej określić i zaznaczyć wszystkie lata Twojego życia, w których sytuacje te miały miejsce.** | | |
| --- | --- | --- |
| 1. Byłeś/-aś świadkiem tego, jak inni dorośli mieszkający w Twoim domu ostro kłócili się z Twoją matką (macochą, babką), obrażali ją lub grozili jej użyciem przemocy.   Zaznacz, ile miałeś/miałaś lat w każdym przypadku, gdy dochodziło do takiego zdarzenia.   \| 1 \| 2 \| 3 \| 4 \| 5 \| 6 \| 7 \| 8 \| 9 \| 10 \| 11 \| 12 \| 13 \| 14 \| 15 \| 16 \| 17 \| 18 \| \| --- \| --- \| --- \| --- \| --- \| --- \| --- \| --- \| --- \| --- \| --- \| --- \| --- \| --- \| --- \| --- \| --- \| --- \| \|  \|  \|  \|  \|  \|  \|  \|  \|  \|  \|  \|  \|  \|  \|  \|  \|  \|  \|   Wskaż, czy odczuwałeś/-aś wówczas bezradność i/lub przerażenie. | 🔿  Tak_1_  ❒ Bezradność | 🔿  Nie_0_  ❒ Przerażenie |
| 1. Byłeś/-aś świadkiem tego, jak inni dorośli mieszkający w Twoim domu ostro kłócili się z Twoim ojcem (ojczymem, dziadkiem), obrażali go lub grozili mu użyciem przemocy.   Zaznacz, ile miałeś/miałaś lat w każdym przypadku, gdy dochodziło do takiego zdarzenia.   \| 1 \| 2 \| 3 \| 4 \| 5 \| 6 \| 7 \| 8 \| 9 \| 10 \| 11 \| 12 \| 13 \| 14 \| 15 \| 16 \| 17 \| 18 \| \| --- \| --- \| --- \| --- \| --- \| --- \| --- \| --- \| --- \| --- \| --- \| --- \| --- \| --- \| --- \| --- \| --- \| --- \| \|  \|  \|  \|  \|  \|  \|  \|  \|  \|  \|  \|  \|  \|  \|  \|  \|  \|  \|   Wskaż, czy odczuwałeś/-aś wówczas bezradność i/lub przerażenie. | 🔿  Tak_1_  ❒ Bezradność | 🔿  Nie_0_  ❒ Przerażenie |
| 1. Widziałeś/-aś, jak inni dorośli mieszkający w Twoim domu popychają, szarpią, biją, rzucają jakimś przedmiotem w Twoją matkę (macochę, babcię).   Zaznacz, ile miałeś/miałaś lat w każdym przypadku, gdy dochodziło do takiego zdarzenia.   \| 1 \| 2 \| 3 \| 4 \| 5 \| 6 \| 7 \| 8 \| 9 \| 10 \| 11 \| 12 \| 13 \| 14 \| 15 \| 16 \| 17 \| 18 \| \| --- \| --- \| --- \| --- \| --- \| --- \| --- \| --- \| --- \| --- \| --- \| --- \| --- \| --- \| --- \| --- \| --- \| --- \| \|  \|  \|  \|  \|  \|  \|  \|  \|  \|  \|  \|  \|  \|  \|  \|  \|  \|  \|   Wskaż, czy odczuwałeś/-aś wówczas bezradność i/lub przerażenie. | 🔿  Tak_1_  ❒ Bezradność | 🔿  Nie_0_  ❒ Przerażenie |
| 1. Widziałeś/-aś, jak inni dorośli mieszkający w Twoim domu popychają, szarpią, biją, rzucają jakimś przedmiotem w Twojego ojca (ojczyma, dziadka).   Zaznacz, ile miałeś/miałaś lat w każdym przypadku, gdy dochodziło do takiego zdarzenia.   \| 1 \| 2 \| 3 \| 4 \| 5 \| 6 \| 7 \| 8 \| 9 \| 10 \| 11 \| 12 \| 13 \| 14 \| 15 \| 16 \| 17 \| 18 \| \| --- \| --- \| --- \| --- \| --- \| --- \| --- \| --- \| --- \| --- \| --- \| --- \| --- \| --- \| --- \| --- \| --- \| --- \| \|  \|  \|  \|  \|  \|  \|  \|  \|  \|  \|  \|  \|  \|  \|  \|  \|  \|  \|   Wskaż, czy odczuwałeś/-aś wówczas bezradność i/lub przerażenie. | 🔿  Tak_1_  ❒ Bezradność | 🔿  Nie_0_  ❒ Przerażenie |

| **Czasem zdarza się, że dzieci w Twoim wieku lub starsze wyrządzają krzywdę,**  **np. prześladują Cię lub znęcają się nad Tobą.**  **Jeśli któraś z poniższych sytuacji zdarzyła się w pierwszych osiemnastu latach Twojego życia,**  **zaznacz TAK.**  **Postaraj się także jak najlepiej określić i zaznaczyć wszystkie lata Twojego życia, w których sytuacje te miały miejsce.** | | |
| --- | --- | --- |
| 1. Używano wobec Ciebie wulgarnego języka, wyzwisk, wypowiadano obraźliwe treści, mówiąc, że jesteś “gruby/-a”, “brzydki/-a”, “głupi/-a” częściej niż kilka razy w roku.   Zaznacz, ile miałeś/miałaś lat w każdym przypadku, gdy dochodziło do takiego zdarzenia.   \| 1 \| 2 \| 3 \| 4 \| 5 \| 6 \| 7 \| 8 \| 9 \| 10 \| 11 \| 12 \| 13 \| 14 \| 15 \| 16 \| 17 \| 18 \| \| --- \| --- \| --- \| --- \| --- \| --- \| --- \| --- \| --- \| --- \| --- \| --- \| --- \| --- \| --- \| --- \| --- \| --- \| \|  \|  \|  \|  \|  \|  \|  \|  \|  \|  \|  \|  \|  \|  \|  \|  \|  \|  \|   Jeśli byłeś/-aś tak traktowany/-a przez osobę, z którą się spotykałeś/-aś (tj. Twojego chłopaka,  dziewczynę, kogoś z kim łączyły Cię relacje towarzyskie, romantyczne lub intymne), wskaż,  ile miałeś/-aś wtedy lat.   \| 1 \| 2 \| 3 \| 4 \| 5 \| 6 \| 7 \| 8 \| 9 \| 10 \| 11 \| 12 \| 13 \| 14 \| 15 \| 16 \| 17 \| 18 \| \| --- \| --- \| --- \| --- \| --- \| --- \| --- \| --- \| --- \| --- \| --- \| --- \| --- \| --- \| --- \| --- \| --- \| --- \| \|  \|  \|  \|  \|  \|  \|  \|  \|  \|  \|  \|  \|  \|  \|  \|  \|  \|  \| | 🔿  Tak_1_  🔿  Tak_1_ | 🔿  Nie_0_  🔿  Nie_0_ |
| 1. Wypowiadano bolesne słowa, które powodowały, że było Ci przykro, czułeś/-aś się zawstydzony/-a lub upokorzony/-a częściej niż kilka razy w roku.   Zaznacz, ile miałeś/miałaś lat w każdym przypadku, gdy dochodziło do takiego zdarzenia.   \| 1 \| 2 \| 3 \| 4 \| 5 \| 6 \| 7 \| 8 \| 9 \| 10 \| 11 \| 12 \| 13 \| 14 \| 15 \| 16 \| 17 \| 18 \| \| --- \| --- \| --- \| --- \| --- \| --- \| --- \| --- \| --- \| --- \| --- \| --- \| --- \| --- \| --- \| --- \| --- \| --- \| \|  \|  \|  \|  \|  \|  \|  \|  \|  \|  \|  \|  \|  \|  \|  \|  \|  \|  \|   Jeśli byłeś/-aś tak traktowany/-a przez osobę, z którą się spotykałeś/-aś (tj. Twojego chłopaka,  dziewczynę, kogoś z kim łączyły Cię relacje towarzyskie, romantyczne lub intymne), wskaż,  ile miałeś/-aś wtedy lat.   \| 1 \| 2 \| 3 \| 4 \| 5 \| 6 \| 7 \| 8 \| 9 \| 10 \| 11 \| 12 \| 13 \| 14 \| 15 \| 16 \| 17 \| 18 \| \| --- \| --- \| --- \| --- \| --- \| --- \| --- \| --- \| --- \| --- \| --- \| --- \| --- \| --- \| --- \| --- \| --- \| --- \| \|  \|  \|  \|  \|  \|  \|  \|  \|  \|  \|  \|  \|  \|  \|  \|  \|  \|  \| | 🔿  Tak_1_    🔿  Tak_1_ | 🔿  Nie_0_  🔿  Nie_0_ |
| 1. Obgadywano Cię za plecami, umieszczano obraźliwe posty w Internecie lub rozpuszczano na Twój temat plotki.   Zaznacz, ile miałeś/miałaś lat w każdym przypadku, gdy dochodziło do takiego zdarzenia.   \| 1 \| 2 \| 3 \| 4 \| 5 \| 6 \| 7 \| 8 \| 9 \| 10 \| 11 \| 12 \| 13 \| 14 \| 15 \| 16 \| 17 \| 18 \| \| --- \| --- \| --- \| --- \| --- \| --- \| --- \| --- \| --- \| --- \| --- \| --- \| --- \| --- \| --- \| --- \| --- \| --- \| \|  \|  \|  \|  \|  \|  \|  \|  \|  \|  \|  \|  \|  \|  \|  \|  \|  \|  \|   Jeśli byłeś/-aś tak traktowany/-a przez osobę, z którą się spotykałeś/-aś (tj. Twojego chłopaka,  dziewczynę, kogoś z kim łączyły Cię relacje towarzyskie, romantyczne lub intymne), wskaż,  ile miałeś/-aś wtedy lat.   \| 1 \| 2 \| 3 \| 4 \| 5 \| 6 \| 7 \| 8 \| 9 \| 10 \| 11 \| 12 \| 13 \| 14 \| 15 \| 16 \| 17 \| 18 \| \| --- \| --- \| --- \| --- \| --- \| --- \| --- \| --- \| --- \| --- \| --- \| --- \| --- \| --- \| --- \| --- \| --- \| --- \| \|  \|  \|  \|  \|  \|  \|  \|  \|  \|  \|  \|  \|  \|  \|  \|  \|  \|  \| | 🔿  Tak_1_    🔿  Tak_1_ | 🔿  Nie_0_  🔿  Nie_0_ |
| 1. Celowo wykluczano Cię z aktywności lub grup.   Zaznacz, ile miałeś/miałaś lat w każdym przypadku, gdy dochodziło do takiego zdarzenia.   \| 1 \| 2 \| 3 \| 4 \| 5 \| 6 \| 7 \| 8 \| 9 \| 10 \| 11 \| 12 \| 13 \| 14 \| 15 \| 16 \| 17 \| 18 \| \| --- \| --- \| --- \| --- \| --- \| --- \| --- \| --- \| --- \| --- \| --- \| --- \| --- \| --- \| --- \| --- \| --- \| --- \| \|  \|  \|  \|  \|  \|  \|  \|  \|  \|  \|  \|  \|  \|  \|  \|  \|  \|  \|   Jeśli byłeś/-aś tak traktowany/-a przez osobę, z którą się spotykałeś/-aś (tj. Twojego chłopaka,  dziewczynę, kogoś z kim łączyły Cię relacje towarzyskie, romantyczne lub intymne), wskaż,  ile miałeś/-aś wtedy lat.   \| 1 \| 2 \| 3 \| 4 \| 5 \| 6 \| 7 \| 8 \| 9 \| 10 \| 11 \| 12 \| 13 \| 14 \| 15 \| 16 \| 17 \| 18 \| \| --- \| --- \| --- \| --- \| --- \| --- \| --- \| --- \| --- \| --- \| --- \| --- \| --- \| --- \| --- \| --- \| --- \| --- \| \|  \|  \|  \|  \|  \|  \|  \|  \|  \|  \|  \|  \|  \|  \|  \|  \|  \|  \| | 🔿  Tak_1_    🔿  Tak_1_ | 🔿  Nie_0_  🔿  Nie_0_ |
| 1. Zachowywano się w sposób, który sprawiał, że obawiałeś/-aś się, że możesz doznać krzywdy fizycznej.   Zaznacz, ile miałeś/miałaś lat w każdym przypadku, gdy dochodziło do takiego zdarzenia.   \| 1 \| 2 \| 3 \| 4 \| 5 \| 6 \| 7 \| 8 \| 9 \| 10 \| 11 \| 12 \| 13 \| 14 \| 15 \| 16 \| 17 \| 18 \| \| --- \| --- \| --- \| --- \| --- \| --- \| --- \| --- \| --- \| --- \| --- \| --- \| --- \| --- \| --- \| --- \| --- \| --- \| \|  \|  \|  \|  \|  \|  \|  \|  \|  \|  \|  \|  \|  \|  \|  \|  \|  \|  \|   Jeśli byłeś/-aś tak traktowany/-a przez osobę, z którą się spotykałeś/-aś (tj. Twojego chłopaka,  dziewczynę, kogoś z kim łączyły Cię relacje towarzyskie, romantyczne lub intymne), wskaż,  ile miałeś/-aś wtedy lat.   \| 1 \| 2 \| 3 \| 4 \| 5 \| 6 \| 7 \| 8 \| 9 \| 10 \| 11 \| 12 \| 13 \| 14 \| 15 \| 16 \| 17 \| 18 \| \| --- \| --- \| --- \| --- \| --- \| --- \| --- \| --- \| --- \| --- \| --- \| --- \| --- \| --- \| --- \| --- \| --- \| --- \| \|  \|  \|  \|  \|  \|  \|  \|  \|  \|  \|  \|  \|  \|  \|  \|  \|  \|  \| | 🔿  Tak_1_    🔿  Tak_1_ | 🔿  Nie_0_  🔿  Nie_0_ |
| 1. Zastraszaniem wymuszano na Tobie oddanie pieniędzy lub Twoich rzeczy.   Zaznacz, ile miałeś/miałaś lat w każdym przypadku, gdy dochodziło do takiego zdarzenia.   \| 1 \| 2 \| 3 \| 4 \| 5 \| 6 \| 7 \| 8 \| 9 \| 10 \| 11 \| 12 \| 13 \| 14 \| 15 \| 16 \| 17 \| 18 \| \| --- \| --- \| --- \| --- \| --- \| --- \| --- \| --- \| --- \| --- \| --- \| --- \| --- \| --- \| --- \| --- \| --- \| --- \| \|  \|  \|  \|  \|  \|  \|  \|  \|  \|  \|  \|  \|  \|  \|  \|  \|  \|  \|   Jeśli byłeś/-aś tak traktowany/-a przez osobę, z którą się spotykałeś/-aś (tj. Twojego chłopaka,  dziewczynę, kogoś z kim łączyły Cię relacje towarzyskie, romantyczne lub intymne), wskaż,  ile miałeś/-aś wtedy lat.   \| 1 \| 2 \| 3 \| 4 \| 5 \| 6 \| 7 \| 8 \| 9 \| 10 \| 11 \| 12 \| 13 \| 14 \| 15 \| 16 \| 17 \| 18 \| \| --- \| --- \| --- \| --- \| --- \| --- \| --- \| --- \| --- \| --- \| --- \| --- \| --- \| --- \| --- \| --- \| --- \| --- \| \|  \|  \|  \|  \|  \|  \|  \|  \|  \|  \|  \|  \|  \|  \|  \|  \|  \|  \| | 🔿  Tak_1_    🔿  Tak_1_ | 🔿  Nie_0_  🔿  Nie_0_ |
| 1. Siłą lub przy pomocy gróźb zmuszano Cię do robienia rzeczy wbrew Twojej woli.   Zaznacz, ile miałeś/miałaś lat w każdym przypadku, gdy dochodziło do takiego zdarzenia.   \| 1 \| 2 \| 3 \| 4 \| 5 \| 6 \| 7 \| 8 \| 9 \| 10 \| 11 \| 12 \| 13 \| 14 \| 15 \| 16 \| 17 \| 18 \| \| --- \| --- \| --- \| --- \| --- \| --- \| --- \| --- \| --- \| --- \| --- \| --- \| --- \| --- \| --- \| --- \| --- \| --- \| \|  \|  \|  \|  \|  \|  \|  \|  \|  \|  \|  \|  \|  \|  \|  \|  \|  \|  \|   Jeśli tak, opisz, jakie to były sytuacje:   \|  \| \| --- \|   Jeśli byłeś/-aś tak traktowany/-a przez osobę, z którą się spotykałeś/-aś (tj. Twojego chłopaka,  dziewczynę, kogoś z kim łączyły Cię relacje towarzyskie, romantyczne lub intymne), wskaż,  ile miałeś/-aś wtedy lat.   \| 1 \| 2 \| 3 \| 4 \| 5 \| 6 \| 7 \| 8 \| 9 \| 10 \| 11 \| 12 \| 13 \| 14 \| 15 \| 16 \| 17 \| 18 \| \| --- \| --- \| --- \| --- \| --- \| --- \| --- \| --- \| --- \| --- \| --- \| --- \| --- \| --- \| --- \| --- \| --- \| --- \| \|  \|  \|  \|  \|  \|  \|  \|  \|  \|  \|  \|  \|  \|  \|  \|  \|  \|  \| | 🔿  Tak_1_    🔿  Tak_1_ | 🔿  Nie_0_  🔿  Nie_0_ |
| 1. Celowo popychano Cię, szarpano, szczypano, uderzano otwartą dłonią lub pięścią bądź kopano.   Zaznacz, ile miałeś/miałaś lat w każdym przypadku, gdy dochodziło do takiego zdarzenia.   \| 1 \| 2 \| 3 \| 4 \| 5 \| 6 \| 7 \| 8 \| 9 \| 10 \| 11 \| 12 \| 13 \| 14 \| 15 \| 16 \| 17 \| 18 \| \| --- \| --- \| --- \| --- \| --- \| --- \| --- \| --- \| --- \| --- \| --- \| --- \| --- \| --- \| --- \| --- \| --- \| --- \| \|  \|  \|  \|  \|  \|  \|  \|  \|  \|  \|  \|  \|  \|  \|  \|  \|  \|  \|   Jeśli byłeś/-aś tak traktowany/-a przez osobę, z którą się spotykałeś/-aś (tj. Twojego chłopaka,  dziewczynę, kogoś z kim łączyły Cię relacje towarzyskie, romantyczne lub intymne), wskaż,  ile miałeś/-aś wtedy lat.   \| 1 \| 2 \| 3 \| 4 \| 5 \| 6 \| 7 \| 8 \| 9 \| 10 \| 11 \| 12 \| 13 \| 14 \| 15 \| 16 \| 17 \| 18 \| \| --- \| --- \| --- \| --- \| --- \| --- \| --- \| --- \| --- \| --- \| --- \| --- \| --- \| --- \| --- \| --- \| --- \| --- \| \|  \|  \|  \|  \|  \|  \|  \|  \|  \|  \|  \|  \|  \|  \|  \|  \|  \|  \| | 🔿  Tak_1_    🔿  Tak_1_ | 🔿  Nie_0_  🔿  Nie_0_ |
| 1. Bito Cię lub w inny sposób krzywdzono tak mocno, że potrzebowałeś/-aś pomocy medycznej.   Zaznacz, ile miałeś/miałaś lat w każdym przypadku, gdy dochodziło do takiego zdarzenia.   \| 1 \| 2 \| 3 \| 4 \| 5 \| 6 \| 7 \| 8 \| 9 \| 10 \| 11 \| 12 \| 13 \| 14 \| 15 \| 16 \| 17 \| 18 \| \| --- \| --- \| --- \| --- \| --- \| --- \| --- \| --- \| --- \| --- \| --- \| --- \| --- \| --- \| --- \| --- \| --- \| --- \| \|  \|  \|  \|  \|  \|  \|  \|  \|  \|  \|  \|  \|  \|  \|  \|  \|  \|  \|   Jeśli byłeś/-aś tak traktowany/-a przez osobę, z którą się spotykałeś/-aś (tj. Twojego chłopaka,  dziewczynę, kogoś z kim łączyły Cię relacje towarzyskie, romantyczne lub intymne), wskaż,  ile miałeś/-aś wtedy lat.   \| 1 \| 2 \| 3 \| 4 \| 5 \| 6 \| 7 \| 8 \| 9 \| 10 \| 11 \| 12 \| 13 \| 14 \| 15 \| 16 \| 17 \| 18 \| \| --- \| --- \| --- \| --- \| --- \| --- \| --- \| --- \| --- \| --- \| --- \| --- \| --- \| --- \| --- \| --- \| --- \| --- \| \|  \|  \|  \|  \|  \|  \|  \|  \|  \|  \|  \|  \|  \|  \|  \|  \|  \|  \| | 🔿  Tak_1_    🔿  Tak_1_ | 🔿  Nie_0_  🔿  Nie_0_ |
| 1. Zmuszano Cię do aktywności seksualnej wbrew Twojej woli.   Zaznacz, ile miałeś/miałaś lat w każdym przypadku, gdy dochodziło do takiego zdarzenia.   \| 1 \| 2 \| 3 \| 4 \| 5 \| 6 \| 7 \| 8 \| 9 \| 10 \| 11 \| 12 \| 13 \| 14 \| 15 \| 16 \| 17 \| 18 \| \| --- \| --- \| --- \| --- \| --- \| --- \| --- \| --- \| --- \| --- \| --- \| --- \| --- \| --- \| --- \| --- \| --- \| --- \| \|  \|  \|  \|  \|  \|  \|  \|  \|  \|  \|  \|  \|  \|  \|  \|  \|  \|  \|   Jeśli byłeś/-aś tak traktowany/-a przez osobę, z którą się spotykałeś/-aś (tj. Twojego chłopaka,  dziewczynę, kogoś z kim łączyły Cię relacje towarzyskie, romantyczne lub intymne), wskaż,  ile miałeś/-aś wtedy lat.   \| 1 \| 2 \| 3 \| 4 \| 5 \| 6 \| 7 \| 8 \| 9 \| 10 \| 11 \| 12 \| 13 \| 14 \| 15 \| 16 \| 17 \| 18 \| \| --- \| --- \| --- \| --- \| --- \| --- \| --- \| --- \| --- \| --- \| --- \| --- \| --- \| --- \| --- \| --- \| --- \| --- \| \|  \|  \|  \|  \|  \|  \|  \|  \|  \|  \|  \|  \|  \|  \|  \|  \|  \|  \| | 🔿  Tak_1_    🔿  Tak_1_ | 🔿  Nie_0_  🔿  Nie_0_ |
| 1. Zmuszano Cię do robienia rzeczy o charakterze seksualnym wbrew Twojej woli.   Zaznacz, ile miałeś/miałaś lat w każdym przypadku, gdy dochodziło do takiego zdarzenia.   \| 1 \| 2 \| 3 \| 4 \| 5 \| 6 \| 7 \| 8 \| 9 \| 10 \| 11 \| 12 \| 13 \| 14 \| 15 \| 16 \| 17 \| 18 \| \| --- \| --- \| --- \| --- \| --- \| --- \| --- \| --- \| --- \| --- \| --- \| --- \| --- \| --- \| --- \| --- \| --- \| --- \| \|  \|  \|  \|  \|  \|  \|  \|  \|  \|  \|  \|  \|  \|  \|  \|  \|  \|  \|   Jeśli byłeś/-aś tak traktowany/-a przez osobę, z którą się spotykałeś/-aś (tj. Twojego chłopaka,  dziewczynę, kogoś z kim łączyły Cię relacje towarzyskie, romantyczne lub intymne), wskaż,  ile miałeś/-aś wtedy lat.   \| 1 \| 2 \| 3 \| 4 \| 5 \|  \| 6 \| 7 \| 8 \| 9 \| 10 \| 11 \| 12 \| 13 \| 14 \| 15 \| 16 \| 17 \| 18 \| \| --- \| --- \| --- \| --- \| --- \| --- \| --- \| --- \| --- \| --- \| --- \| --- \| --- \| --- \| --- \| --- \| --- \| --- \| --- \| \|  \|  \|  \|  \|  \|  \|  \|  \|  \|  \|  \|  \|  \|  \|  \|  \|  \|  \|  \| | 🔿  Tak_1_    🔿  Tak_1_ | 🔿  Nie_0_  🔿  Nie_0_ |

| **Wskaż, które z poniższych zdarzeń miało miejsce w Twoim dzieciństwie (przez pierwsze 18 lat życia).**    **Postaraj się także jak najlepiej określić i zaznaczyć wszystkie lata Twojego życia, w których sytuacje te miały miejsce.** | | |
| --- | --- | --- |
| 1. Czułeś/-aś, że Twoja matka lub inna ważna dla Ciebie kobieta pełniąca rolę matki była obecna w domu, jednak pozostawała dla Ciebie niedostępna emocjonalnie z różnych powodów,   takich jak używanie narkotyków lub alkoholu, pracoholizm, romanse, czy też beztroskie  skupienie się na własnych celach.  Zaznacz, ile miałeś/miałaś lat w każdym przypadku, gdy dochodziło do takiego zdarzenia.   \| 1 \| 2 \| 3 \| 4 \| 5 \| 6 \| 7 \| 8 \| 9 \| 10 \| 11 \| 12 \| 13 \| 14 \| 15 \| 16 \| 17 \| 18 \| \| --- \| --- \| --- \| --- \| --- \| --- \| --- \| --- \| --- \| --- \| --- \| --- \| --- \| --- \| --- \| --- \| --- \| --- \| \|  \|  \|  \|  \|  \|  \|  \|  \|  \|  \|  \|  \|  \|  \|  \|  \|  \|  \| | 🔿  Tak_1_ | 🔿  Nie_0_ |
| 1. Czułeś/-aś, że Twój ojciec lub inny ważny dla Ciebie mężczyzna pełniący rolę ojca był obecny w domu, jednak pozostawał dla Ciebie niedostępny emocjonalnie z różnych powodów, takich jak używanie narkotyków lub alkoholu, pracoholizm, romanse, czy też beztroskie skupienie się na własnych celach.   Zaznacz, ile miałeś/miałaś lat w każdym przypadku, gdy dochodziło do takiego zdarzenia.   \| 1 \| 2 \| 3 \| 4 \| 5 \| 6 \| 7 \| 8 \| 9 \| 10 \| 11 \| 12 \| 13 \| 14 \| 15 \| 16 \| 17 \| 18 \| \| --- \| --- \| --- \| --- \| --- \| --- \| --- \| --- \| --- \| --- \| --- \| --- \| --- \| --- \| --- \| --- \| --- \| --- \| \|  \|  \|  \|  \|  \|  \|  \|  \|  \|  \|  \|  \|  \|  \|  \|  \|  \|  \| | 🔿  Tak_1_ | 🔿  Nie_0_ |
| 1. Czułeś/-aś, że Twoja matka lub inna ważna dla Ciebie kobieta pełniąca rolę matki była niedostępna emocjonalnie z różnych powodów, takich jak odbywanie służby wojskowej, opieka nad chorym krewnym, kontynuacja nauki, zajmowanie się sprawami zawodowymi.   Zaznacz, ile miałeś/miałaś lat w każdym przypadku, gdy dochodziło do takiego zdarzenia.   \| 1 \| 2 \| 3 \| 4 \| 5 \| 6 \| 7 \| 8 \| 9 \| 10 \| 11 \| 12 \| 13 \| 14 \| 15 \| 16 \| 17 \| 18 \| \| --- \| --- \| --- \| --- \| --- \| --- \| --- \| --- \| --- \| --- \| --- \| --- \| --- \| --- \| --- \| --- \| --- \| --- \| \|  \|  \|  \|  \|  \|  \|  \|  \|  \|  \|  \|  \|  \|  \|  \|  \|  \|  \| | 🔿  Tak_1_ | 🔿  Nie_0_ |
| 1. Czułeś/-aś, że Twój ojciec lub inny ważny dla Ciebie mężczyzna pełniący rolę ojca był niedostępny emocjonalnie z różnych powodów, takich jak odbywanie służby wojskowej, opieka nad chorym krewnym, kontynuacja nauki, zajmowanie się sprawami zawodowymi.   Zaznacz, ile miałeś/miałaś lat w każdym przypadku, gdy dochodziło do takiego zdarzenia.   \| 1 \| 2 \| 3 \| 4 \| 5 \| 6 \| 7 \| 8 \| 9 \| 10 \| 11 \| 12 \| 13 \| 14 \| 15 \| 16 \| 17 \| 18 \| \| --- \| --- \| --- \| --- \| --- \| --- \| --- \| --- \| --- \| --- \| --- \| --- \| --- \| --- \| --- \| --- \| --- \| --- \| \|  \|  \|  \|  \|  \|  \|  \|  \|  \|  \|  \|  \|  \|  \|  \|  \|  \|  \| | 🔿  Tak_1_ | 🔿  Nie_0_ |
| 1. Było Ci bardzo ciężko zadowolić rodzica lub inną ważną dla Ciebie osobę pełniącą rolę rodzica.   Zaznacz, ile miałeś/miałaś lat w każdym przypadku, gdy dochodziło do takiego zdarzenia.   \| 1 \| 2 \| 3 \| 4 \| 5 \| 6 \| 7 \| 8 \| 9 \| 10 \| 11 \| 12 \| 13 \| 14 \| 15 \| 16 \| 17 \| 18 \| \| --- \| --- \| --- \| --- \| --- \| --- \| --- \| --- \| --- \| --- \| --- \| --- \| --- \| --- \| --- \| --- \| --- \| --- \| \|  \|  \|  \|  \|  \|  \|  \|  \|  \|  \|  \|  \|  \|  \|  \|  \|  \|  \| | 🔿  Tak_1_ | 🔿  Nie_0_ |
| 1. Rodzic lub inna ważna osoba pełniąca rolę rodzica nie miał czasu lub chęci żeby z Tobą rozmawiać.   Zaznacz, ile miałeś/miałaś lat w każdym przypadku, gdy dochodziło do takiego zdarzenia.   \| 1 \| 2 \| 3 \| 4 \| 5 \| 6 \| 7 \| 8 \| 9 \| 10 \| 11 \| 12 \| 13 \| 14 \| 15 \| 16 \| 17 \| 18 \| \| --- \| --- \| --- \| --- \| --- \| --- \| --- \| --- \| --- \| --- \| --- \| --- \| --- \| --- \| --- \| --- \| --- \| --- \| \|  \|  \|  \|  \|  \|  \|  \|  \|  \|  \|  \|  \|  \|  \|  \|  \|  \|  \| | 🔿  Tak_1_ | 🔿  Nie_0_ |
| 1. Co najmniej jeden z członków Twojej rodziny sprawiał, że czułeś/-aś się kochany/-a.   Zaznacz, ile miałeś/miałaś lat w każdym przypadku, gdy dochodziło do takiego zdarzenia.   \| 1 \| 2 \| 3 \| 4 \| 5 \| 6 \| 7 \| 8 \| 9 \| 10 \| 11 \| 12 \| 13 \| 14 \| 15 \| 16 \| 17 \| 18 \| \| --- \| --- \| --- \| --- \| --- \| --- \| --- \| --- \| --- \| --- \| --- \| --- \| --- \| --- \| --- \| --- \| --- \| --- \| \|  \|  \|  \|  \|  \|  \|  \|  \|  \|  \|  \|  \|  \|  \|  \|  \|  \|  \|     Kto to był? (np. matka, ciotka, dziadek ze strony matki)   \|  \| \| --- \| | 🔿  Tak_1_ | 🔿  Nie_0_ |
| 1. Co najmniej jeden z członków Twojej rodziny sprawiał, że czułeś/-aś się ważny/-a lub wyjątkowy/-a.   Zaznacz, ile miałeś/miałaś lat w każdym przypadku, gdy dochodziło do takiego zdarzenia.   \| 1 \| 2 \| 3 \| 4 \| 5 \| 6 \| 7 \| 8 \| 9 \| 10 \| 11 \| 12 \| 13 \| 14 \| 15 \| 16 \| 17 \| 18 \| \| --- \| --- \| --- \| --- \| --- \| --- \| --- \| --- \| --- \| --- \| --- \| --- \| --- \| --- \| --- \| --- \| --- \| --- \| \|  \|  \|  \|  \|  \|  \|  \|  \|  \|  \|  \|  \|  \|  \|  \|  \|  \|  \|   Kto to był? (np. matka, ciotka, dziadek ze strony matki)   \|  \| \| --- \| | 🔿  Tak_1_ | 🔿  Nie_0_ |
| 1. Co najmniej jeden z członków Twojej rodziny opiekował się Tobą i chronił Cię.   Zaznacz, ile miałeś/miałaś lat w każdym przypadku, gdy dochodziło do takiego zdarzenia.   \| 1 \| 2 \| 3 \| 4 \| 5 \| 6 \| 7 \| 8 \| 9 \| 10 \| 11 \| 12 \| 13 \| 14 \| 15 \| 16 \| 17 \| 18 \| \| --- \| --- \| --- \| --- \| --- \| --- \| --- \| --- \| --- \| --- \| --- \| --- \| --- \| --- \| --- \| --- \| --- \| --- \| \|  \|  \|  \|  \|  \|  \|  \|  \|  \|  \|  \|  \|  \|  \|  \|  \|  \|  \|     Kto to był? (np. matka, ciotka, dziadek ze strony matki)   \|  \| \| --- \| | 🔿  Tak_1_ | 🔿  Nie_0_ |
| 1. Co najmniej jeden członek Twojej rodziny zabierał Cię do lekarza lub szpitala, jeśli zaistniała taka potrzeba.   Zaznacz, ile miałeś/miałaś lat w każdym przypadku, gdy dochodziło do takiego zdarzenia.   \| 1 \| 2 \| 3 \| 4 \| 5 \| 6 \| 7 \| 8 \| 9 \| 10 \| 11 \| 12 \| 13 \| 14 \| 15 \| 16 \| 17 \| 18 \| \| --- \| --- \| --- \| --- \| --- \| --- \| --- \| --- \| --- \| --- \| --- \| --- \| --- \| --- \| --- \| --- \| --- \| --- \| \|  \|  \|  \|  \|  \|  \|  \|  \|  \|  \|  \|  \|  \|  \|  \|  \|  \|  \|     Kto to był? (np. matka, ciotka, dziadek ze strony matki)   \|  \| \| --- \| | 🔿  Tak_1_ | 🔿  Nie_0_ |
| 1. Co najmniej jeden z członków Twojej rodziny pomagał Ci przy odrabianiu lekcji lub przygotowaniach do szkoły.   Zaznacz, ile miałeś/miałaś lat w każdym przypadku, gdy dochodziło do takiego zdarzenia.   \| 1 \| 2 \| 3 \| 4 \| 5 \| 6 \| 7 \| 8 \| 9 \| 10 \| 11 \| 12 \| 13 \| 14 \| 15 \| 16 \| 17 \| 18 \| \| --- \| --- \| --- \| --- \| --- \| --- \| --- \| --- \| --- \| --- \| --- \| --- \| --- \| --- \| --- \| --- \| --- \| --- \| \|  \|  \|  \|  \|  \|  \|  \|  \|  \|  \|  \|  \|  \|  \|  \|  \|  \|  \| | 🔿  Tak_1_ | 🔿  Nie_0_ |

| **Wskaż, czy poniższe stwierdzenia są prawdziwe w odniesieniu do Ciebie i Twojej rodziny w okresie**  **Twojego dzieciństwa. Podaj swój wiek w chwili, gdy czułeś, że te sytuacje miały miejsce.**  **Zaznacz, ile miałeś/miałaś lat w każdym przypadku, gdy dochodziło do takiego zdarzenia.** | | |
| --- | --- | --- |
| 1. Brakowało Ci jedzenia.   Zaznacz, ile miałeś/miałaś lat w każdym przypadku, gdy dochodziło do takiego zdarzenia.   \| 1 \| 2 \| 3 \| 4 \| 5 \| 6 \| 7 \| 8 \| 9 \| 10 \| 11 \| 12 \| 13 \| 14 \| 15 \| 16 \| 17 \| 18 \| \| --- \| --- \| --- \| --- \| --- \| --- \| --- \| --- \| --- \| --- \| --- \| --- \| --- \| --- \| --- \| --- \| --- \| --- \| \|  \|  \|  \|  \|  \|  \|  \|  \|  \|  \|  \|  \|  \|  \|  \|  \|  \|  \| | 🔿  Tak_1_ | 🔿  Nie_0_ |
| 1. Musiałeś/-aś chodzić w brudnych ubraniach.   Zaznacz, ile miałeś/miałaś lat w każdym przypadku, gdy dochodziło do takiego zdarzenia.   \| 1 \| 2 \| 3 \| 4 \| 5 \| 6 \| 7 \| 8 \| 9 \| 10 \| 11 \| 12 \| 13 \| 14 \| 15 \| 16 \| 17 \| 18 \| \| --- \| --- \| --- \| --- \| --- \| --- \| --- \| --- \| --- \| --- \| --- \| --- \| --- \| --- \| --- \| --- \| --- \| --- \| \|  \|  \|  \|  \|  \|  \|  \|  \|  \|  \|  \|  \|  \|  \|  \|  \|  \|  \| | 🔿  Tak_1_ | 🔿  Nie_0_ |
| 1. Pozostawiano Cię bez opieki, gdy byłeś/-aś w wieku lub w sytuacjach wymagających nadzoru   osób starszych.  Zaznacz, ile miałeś/miałaś lat w każdym przypadku, gdy dochodziło do takiego zdarzenia.   \| 1 \| 2 \| 3 \| 4 \| 5 \| 6 \| 7 \| 8 \| 9 \| 10 \| 11 \| 12 \| 13 \| 14 \| 15 \| 16 \| 17 \| 18 \| \| --- \| --- \| --- \| --- \| --- \| --- \| --- \| --- \| --- \| --- \| --- \| --- \| --- \| --- \| --- \| --- \| --- \| --- \| \|  \|  \|  \|  \|  \|  \|  \|  \|  \|  \|  \|  \|  \|  \|  \|  \|  \|  \| | 🔿  Tak_1_ | 🔿  Nie_0_ |
| 1. Miałeś/-aś poczucie, że musisz przejmować obowiązki dorosłych.   Zaznacz, ile miałeś/miałaś lat w każdym przypadku, gdy dochodziło do takiego zdarzenia.   \| 1 \| 2 \| 3 \| 4 \| 5 \| 6 \| 7 \| 8 \| 9 \| 10 \| 11 \| 12 \| 13 \| 14 \| 15 \| 16 \| 17 \| 18 \| \| --- \| --- \| --- \| --- \| --- \| --- \| --- \| --- \| --- \| --- \| --- \| --- \| --- \| --- \| --- \| --- \| --- \| --- \| \|  \|  \|  \|  \|  \|  \|  \|  \|  \|  \|  \|  \|  \|  \|  \|  \|  \|  \| | 🔿  Tak_1_ | 🔿  Nie_0_ |
| 1. Miałeś/-aś poczucie, że Twoja rodzina boryka się ze znacznymi kłopotami finansowymi.   Zaznacz, ile miałeś/miałaś lat w każdym przypadku, gdy dochodziło do takiego zdarzenia.   \| 1 \| 2 \| 3 \| 4 \| 5 \| 6 \| 7 \| 8 \| 9 \| 10 \| 11 \| 12 \| 13 \| 14 \| 15 \| 16 \| 17 \| 18 \| \| --- \| --- \| --- \| --- \| --- \| --- \| --- \| --- \| --- \| --- \| --- \| --- \| --- \| --- \| --- \| --- \| --- \| --- \| \|  \|  \|  \|  \|  \|  \|  \|  \|  \|  \|  \|  \|  \|  \|  \|  \|  \|  \| | 🔿  Tak_1_ | 🔿  Nie_0_ |
| 1. Co najmniej jedna osoba utrzymywała przed Tobą w tajemnicy ważne sprawy lub fakty.   Zaznacz, ile miałeś/miałaś lat w każdym przypadku, gdy dochodziło do takiego zdarzenia.   \| 1 \| 2 \| 3 \| 4 \| 5 \| 6 \| 7 \| 8 \| 9 \| 10 \| 11 \| 12 \| 13 \| 14 \| 15 \| 16 \| 17 \| 18 \| \| --- \| --- \| --- \| --- \| --- \| --- \| --- \| --- \| --- \| --- \| --- \| --- \| --- \| --- \| --- \| --- \| --- \| --- \| \|  \|  \|  \|  \|  \|  \|  \|  \|  \|  \|  \|  \|  \|  \|  \|  \|  \|  \| | 🔿  Tak_1_ | 🔿  Nie_0_ |
| 1. Twoi rodzice pozostawali w separacji.   Zaznacz, ile miałeś/miałaś lat w każdym przypadku, gdy dochodziło do takiego zdarzenia.   \| 1 \| 2 \| 3 \| 4 \| 5 \| 6 \| 7 \| 8 \| 9 \| 10 \| 11 \| 12 \| 13 \| 14 \| 15 \| 16 \| 17 \| 18 \| \| --- \| --- \| --- \| --- \| --- \| --- \| --- \| --- \| --- \| --- \| --- \| --- \| --- \| --- \| --- \| --- \| --- \| --- \| \|  \|  \|  \|  \|  \|  \|  \|  \|  \|  \|  \|  \|  \|  \|  \|  \|  \|  \| | 🔿  Tak_1_ | 🔿  Nie_0_ |
| 1. Członkowie Twojej rodziny troszczyli się o siebie nawzajem.   Zaznacz, ile miałeś/miałaś lat w każdym przypadku, gdy dochodziło do takiego zdarzenia.   \| 1 \| 2 \| 3 \| 4 \| 5 \| 6 \| 7 \| 8 \| 9 \| 10 \| 11 \| 12 \| 13 \| 14 \| 15 \| 16 \| 17 \| 18 \| \| --- \| --- \| --- \| --- \| --- \| --- \| --- \| --- \| --- \| --- \| --- \| --- \| --- \| --- \| --- \| --- \| --- \| --- \| \|  \|  \|  \|  \|  \|  \|  \|  \|  \|  \|  \|  \|  \|  \|  \|  \|  \|  \| | 🔿  Tak_1_ | 🔿  Nie_0_ |
| 1. Członków twojej rodziny łączyła bliska więź.   Zaznacz, ile miałeś/miałaś lat w każdym przypadku, gdy dochodziło do takiego zdarzenia.   \| 1 \| 2 \| 3 \| 4 \| 5 \| 6 \| 7 \| 8 \| 9 \| 10 \| 11 \| 12 \| 13 \| 14 \| 15 \| 16 \| 17 \| 18 \| \| --- \| --- \| --- \| --- \| --- \| --- \| --- \| --- \| --- \| --- \| --- \| --- \| --- \| --- \| --- \| --- \| --- \| --- \| \|  \|  \|  \|  \|  \|  \|  \|  \|  \|  \|  \|  \|  \|  \|  \|  \|  \|  \| | 🔿  Tak_1_ | 🔿  Nie_0_ |
| 1. Twoja rodzina była źródłem siły i wsparcia.   Zaznacz, ile miałeś/miałaś lat w każdym przypadku, gdy dochodziło do takiego zdarzenia   \| 1 \| 2 \| 3 \| 4 \| 5 \| 6 \| 7 \| 8 \| 9 \| 10 \| 11 \| 12 \| 13 \| 14 \| 15 \| 16 \| 17 \| 18 \| \| --- \| --- \| --- \| --- \| --- \| --- \| --- \| --- \| --- \| --- \| --- \| --- \| --- \| --- \| --- \| --- \| --- \| --- \| \|  \|  \|  \|  \|  \|  \|  \|  \|  \|  \|  \|  \|  \|  \|  \|  \|  \|  \| | 🔿  Tak_1_ | 🔿  Nie_0_ |
